# Integrative perspectives on electroacupuncture modulation of vagal–cholinergic and neuro–immune–metabolic regulation in long COVID

**DOI:** 10.3389/fnint.2026.1775007

**Published:** 2026-06-05

**Authors:** Liwen Wang, Xiaoyang Hu, Dongning Yan

**Affiliations:** 1 Graduate School, Heilongjiang University of Chinese Medicine, Harbin, Heilongjiang, China; 2 Basic Medical College, Heilongjiang University of Chinese Medicine, Harbin, Heilongjiang, China; 3 Accreditation Center of TCM Physician, National Administration of Traditional Chinese Medicine, Beijing, China

**Keywords:** electroacupuncture, long COVID, vagus nerve, cholinergic anti-inflammatory pathway (CAP), α7-type nicotinic acetylcholine receptors (α7nAChRs), AMPK–SIRT1–PGC-1α, neural–immune–metabolic integration

## Abstract

Long COVID is increasingly recognized as a multisystem condition involving persistent inflammation, autonomic dysregulation, and metabolic disturbance. The vagus nerve–mediated cholinergic anti-inflammatory pathway (CAP) provides a biologically plausible link between neural regulation and immune homeostasis, while metabolic pathways involving AMP-activated protein kinase (AMPK), sirtuin 1 (SIRT1), and peroxisome proliferator-activated receptor gamma coactivator-1α (PGC-1α) are closely related to mitochondrial function and energy balance. In this review, we synthesize evidence from neuroscience, immunology, and metabolic research to investigate how electroacupuncture (EA) may modulate vagal-cholinergic signaling and the downstream inflammatory and metabolic processes associated with long COVID. Experimental studies indicate that EA can influence CAP-related mechanisms, including α7 nicotinic acetylcholine receptor (α7nAChR)-mediated inhibition of NF-κB/NLRP3-related inflammatory signaling, and may also regulate AMPK–SIRT1–PGC-1α-associated metabolic pathways. Although clinical evidence is more indirect, it suggests that electroacupuncture may affect autonomic function, inflammatory markers, symptom burden, and neurophysiological regulation. To support a balanced interpretation, we organize the evidence in this review into a framework based on levels of evidence, which distinguishes direct preclinical findings from indirect clinical indicators and associations used to generate hypotheses. This framework highlights the potential convergence of vagal–cholinergic anti-inflammatory regulation and metabolic recovery pathways, while recognizing that several proposed connections—particularly those linking CAP-related signaling to improvements in long COVID symptoms—require further validation. Overall, this review provides a structured basis for future mechanistic studies and phenotype-oriented clinical trials evaluating EA as a neuromodulatory strategy for long COVID and related chronic inflammatory conditions.

## 1 Introduction

Long COVID (also known as post-COVID syndrome) is generally regarded as a multisystem condition characterized by persistent or recurrent symptoms following acute SARS-CoV-2 infection. Clinical data from various studies indicate marked heterogeneity in its manifestations, encompassing common symptoms such as persistent fatigue, shortness of breath, cognitive impairment (commonly termed “brain fog”), palpitations, and sleep disturbances, as well as gastrointestinal discomfort and emotional changes (Gusev and Sarapultsev, 2023; Malekmohammad et al., 2021). These symptoms often persist for months, exerting sustained impacts on patients’ daily functioning and overall quality of life.

Beyond symptom persistence, an increasing number of studies associate long COVID with chronic low-grade inflammation, immune–metabolic dysregulation, and autonomic imbalance (Moore et al., 2023; Müller and Di Benedetto, 2024; Trautmann, 2025; Xiao et al., 2021). Persistent viral antigens, infection-related tissue injury, and maladaptive immune responses may collectively contribute to the maintenance of pro-inflammatory signaling (Gupta et al., 2025; Michalak et al., 2025), while disturbances in energy metabolism and oxidative stress may further amplify these inflammatory processes (Hotamisligil, 2017; Proal and VanElzakker, 2021; Su et al., 2022a). Importantly, these abnormalities tend to co-occur and reinforce one another rather than acting in isolation, gradually forming interrelated pathological patterns. Although understanding of individual components continues to advance, how these mechanistic elements are integrated at a systemic level remains an unresolved issue.

The vagus nerve–mediated cholinergic anti-inflammatory pathway (CAP) is widely recognized as a key regulatory axis linking neural activity with immune and metabolic homeostasis (Cao et al., 2024; Koopman et al., 2016). Through this pathway, acetylcholine released from vagal efferents modulates immune cell activity and constrains excessive inflammatory responses. Experimental and clinical studies have shown that neuromodulatory interventions targeting vagal function can influence CAP-related signaling. Among these approaches, electroacupuncture (EA) has been increasingly investigated as a controllable form of neuromodulation. However, how EA may simultaneously engage neural, immune, and metabolic networks—particularly in the context of long COVID—has not yet been systematically integrated.

In this review, we organize and synthesize evidence from animal studies, clinical investigations, and research in neuroimmunology and cellular energy regulation to explore how EA-related neuromodulation may interact with vagal regulatory pathways. Conceptual models, including the EA–CAP–AMPK–SIRT1–PGC-1α axis, Neural Resonance Modulation (NRM), and neuro–immune–metabolic (NIM) coupling, are used as integrative tools to facilitate interpretation of existing findings rather than to assert definitive mechanisms. Together, these perspectives aim to provide a structured framework for understanding the multisystem regulatory effects reported in EA studies, with relevance to long COVID and other chronic inflammatory conditions.

### 1.1 Background

According to the World Health Organization (WHO), post COVID-19 condition, also known as long COVID, is characterized by symptoms that usually occur within three months after initial SARS-CoV-2 infection, last for at least two months, and cannot be explained by an alternative diagnosis (World Health Organization, 2021). This definition emphasizes both symptom persistence and diagnostic exclusion in identifying long COVID. Updated WHO information indicates that approximately 6 in 100 people with COVID-19 develop post COVID-19 condition, although estimates vary substantially across populations and study designs (World Health Organization, 2025). A recent global systematic review and meta-analysis including studies searched up to 2024 further reported that long COVID remains common worldwide, with prevalence estimates varying by follow-up duration, region, symptom definition, and study population (Hou et al., 2025). These data underscore the continuing public health relevance of long COVID and the need to clarify mechanisms underlying its persistent multisystem manifestations.

The vagus nerve plays a pivotal role in maintaining physiological homeostasis by establishing bidirectional communication between the central nervous system and peripheral organs. Among its most extensively studied efferent regulatory mechanisms is the CAP, in which acetylcholine released by vagal fibers acts on α7 nicotinic acetylcholine receptors (α7nAChRs) expressed on immune cells, thereby inhibiting pro-inflammatory cytokine production mediated by nuclear factor kappa-B (NF-κB) (Bonaz et al., 2020; Zhan et al., 2025). This neuroimmune feedback mechanism enables dynamic regulation of inflammatory responses across diverse physiological and pathological contexts.

Beyond immunoregulation, CAP-related signaling has also been linked to cellular metabolic control. The AMPK–SIRT1–PGC-1α axis plays a central role in mitochondrial biogenesis, oxidative stress regulation, and energy homeostasis, processes that are closely intertwined with inflammatory signaling. Disruption of these coupled pathways may therefore contribute to the persistence of inflammation and metabolic dysfunction in chronic inflammatory states, including long COVID.

EA, as a non-pharmacological neuromodulation technique, offers the advantage of relatively well-defined stimulation parameters and high reproducibility in experimental and clinical studies. Animal studies have demonstrated that EA applied to commonly used acupoints such as ST36 (Zusanli) and PC6 (Neiguan) can enhance vagal efferent activity, promote acetylcholine release, and modulate α7nAChR expression, accompanied by inhibition of NF-κB and NLRP3 inflammasome-related signaling pathways (Benghanem et al., 2022; Cao et al., 2024; Kathuria et al., 2022; Liu et al., 2021; Liu F. et al., 2024; Wei J. et al., 2025). Correspondingly, clinical studies have reported improvements in heart rate variability parameters, selected metabolic indicators, and systemic inflammatory markers following EA intervention (Kathuria et al., 2022; Liu et al., 2021; Liu F. et al., 2024; Wei J. et al., 2025). Although the precise mechanisms require further clarification, EA is increasingly considered a form of neuromodulation that engages vagal regulatory pathways.

### 1.2 Scope and conceptual framework of this review

Recent studies have reported reduced vagal nerve activity, decreased acetylcholine availability, and downregulated α7nAChR expression in individuals with long COVID (Camici et al., 2024; Giunta et al., 2024; Liu et al., 2020; Liu F. et al., 2024; Popa et al., 2025; Rangon et al., 2020; Yu and Kong, 2023). These findings suggest impaired vagal–parasympathetic reflex function, potentially limiting the effectiveness of CAP-mediated anti-inflammatory signaling. In this review, we use the term “CAP hyporesponsiveness” as a provisional mechanistic descriptor to describe this pattern of reduced responsiveness within the vagal–cholinergic regulatory axis, not as a validated clinical diagnosis.

Based on this conceptualization, the present review synthesizes evidence from animal models, clinical studies, and neurophysiological and metabolic research to examine how EA-related neuromodulation may influence vagus nerve–CAP function and broader neuro–immune–metabolic coordination. By organizing existing findings within integrative frameworks such as the EA–CAP–AMPK–SIRT1–PGC-1α model, NRM, and NIM coupling, we aim to provide a systems-oriented perspective to guide future experimental validation and translational research.

## 2 Pathophysiological basis of long COVID: inflammatory, autonomic, and metabolic disruption

### 2.1 Persistent inflammatory activation and immune dysregulation

Long COVID patients typically exhibit persistent post-infection inflammatory responses characterized by sustained elevation of IL-6, TNF-α, and IL-1β, alongside chronic activation of the monocyte-macrophage system (Amro et al., 2022; Kemper et al., 2023; Smith et al., 2023; Su et al., 2022b). Viral remnants and tissue damage prevent the restoration of normal immune homeostasis (Moradi et al., 2022). Throughout this process, macrophages persistently maintain an M1-dominant state, continuously producing inflammatory mediators (Al-Aly et al., 2022; Su et al., 2022b), while impaired regulatory T cell function further impedes the resolution of inflammation (Smith et al., 2023).

This persistent low-grade inflammatory environment promotes endothelial dysfunction, coagulation activation, and oxidative stress (Mroueh et al., 2024), forming a positive feedback loop that exacerbates microvascular injury and systemic dysfunction (Karnatam et al., 2023; Sarıkaya et al., 2023). This chronic inflammatory burden not only drives organ-specific pathologies (Meng et al., 2022; Moradi et al., 2022) but also disrupts autonomic regulation and cellular metabolic programming through cytokine-driven signaling networks (Nishimura et al., 2020; Smith et al., 2023), resulting in the multisystem dysregulation observed in long COVID (Smith et al., 2023).

### 2.2 Autonomic dysfunction and reduced vagal activity

A significant proportion of long COVID patients exhibit marked autonomic imbalance, characterized by persistent sympathetic overactivation and reduced parasympathetic (vagus nerve) tone (Jiménez-González et al., 2023; Nishimura et al., 2020). HRV assessments typically reveal reduced high-frequency (HF) components and elevated low-frequency/high-frequency (LF/HF) ratios, indicating a shift toward sympathetic dominance in the autonomic nervous system (Dani et al., 2021; Riggan et al., 2021; Smith et al., 2021). At the central level, impaired function within the supranuclear bundle-medullary vagus nerve-hypothalamic network disrupts autonomic dynamic regulation (Mhazo and Maponga, 2022; Witvrouwen et al., 2021). Excessive sympathetic signaling amplifies proinflammatory pathways via the norepinephrine-β2 adrenergic receptor axis (Ayeldeen et al., 2024), while diminished vagal activity weakens cholinergic anti-inflammatory feedback mechanisms, impairing cytokine function (Seufert and Napier, 2023).

Clinically, this imbalance manifests as palpitations, anxiety, sleep disturbances, orthostatic intolerance, and fatigue. Functional neuroimaging studies reveal reduced metabolic activity in brainstem and limbic autonomic centers (Guedj et al., 2021; Tay et al., 2023), further corroborating these findings. Reduced HRV is widely recognized as an indirect yet reliable indicator of diminished autonomic activity (Zhu and Liu, 2025). The resulting autonomic dysfunction not only reflects chronic inflammation’s impact on neural circuits but also amplifies inflammatory signaling through neuroimmune pathways. This suggests that autonomic dysfunction serves both as a hallmark and a driver of the autonomic hypofunction syndrome observed in long COVID.

### 2.3 Immunometabolic reprogramming and mitochondrial dysfunction

Alongside inflammation and autonomic dysfunction, long COVID is frequently accompanied by characteristic metabolic reprogramming (Adilović et al., 2025; Wu et al., 2025). Under persistent inflammatory stimulation, immune cells shift from oxidative phosphorylation to aerobic glycolysis to meet the energy demands of chronic immune activation (Guarnieri et al., 2024; Su et al., 2022b). Although initially adaptive, prolonged reliance on glycolysis impairs ATP production, leads to lactic acid accumulation, and causes progressive mitochondrial dysfunction (Guarnieri et al., 2024). Excessive reactive oxygen species (ROS) generation further damages mitochondrial DNA and membrane integrity, exacerbating oxidative stress and weakening cellular resilience (Semo et al., 2025).

Regulatory factors of core metabolism, including AMPK, SIRT1, and PGC-1α, also become inhibited, leading to impaired mitochondrial biogenesis, reduced antioxidant defense capacity, and disrupted energy homeostasis (Guarnieri et al., 2024; Semo et al., 2025). These metabolic abnormalities constrain tissue repair capacity and neural regulation of inflammation while disrupting neuro-immune coupling mechanisms (Guarnieri et al., 2024; Robles et al., 2023). Collectively, such metabolic-mitochondrial dysfunction exacerbates the systemic dysregulation characteristic of long COVID and interacts with autonomic nervous and inflammatory pathways to perpetuate chronic disease progression (Tsilingiris et al., 2023).

### 2.4 Summary: an integrated model of inflammation, autonomic dysregulation, and metabolic disturbance

Inflammation, autonomic imbalance, and metabolic dysfunction frequently co-occur in long COVID and may form a self-reinforcing pathological triad. Persistent inflammatory activation can affect both central and peripheral autonomic circuits through cytokine signaling, oxidative stress, and endothelial or microvascular injury, thereby disturbing neurotransmitter release and shifting autonomic balance toward sympathetic predominance and reduced vagal tone. Reduced vagal activity, in turn, may weaken cholinergic anti-inflammatory feedback, limiting α7nAChR-mediated suppression of NF-κB/NLRP3-related inflammatory signaling and allowing cytokine activity to persist.

Metabolic dysfunction provides a third reinforcing component of this cycle. Mitochondrial impairment, reduced ATP production, and excessive ROS generation can compromise cellular repair capacity and increase inflammatory susceptibility. At the same time, persistent inflammation and autonomic dysregulation may further disrupt AMPK–SIRT1–PGC-1α-related metabolic control, thereby reducing metabolic resilience and sustaining fatigue, exercise intolerance, and multisystem vulnerability.

From this perspective, we conceptualize these interconnected processes as the “inflammation–autonomic–metabolic pathological triad.” This framework helps explain why symptoms may persist and why dysfunction can evolve into a multisystemic condition rather than remaining confined to a single organ system. The key regulatory element in this cycle may be attenuation of vagus nerve-mediated cholinergic anti-inflammatory signaling, leading to reduced CAP-related feedback and impaired regulation of cytokine responses. Examining long COVID through this triadic framework provides a mechanistic basis for the subsequent discussion of EA as a potential neuromodulatory intervention.

**FIGURE 1:**
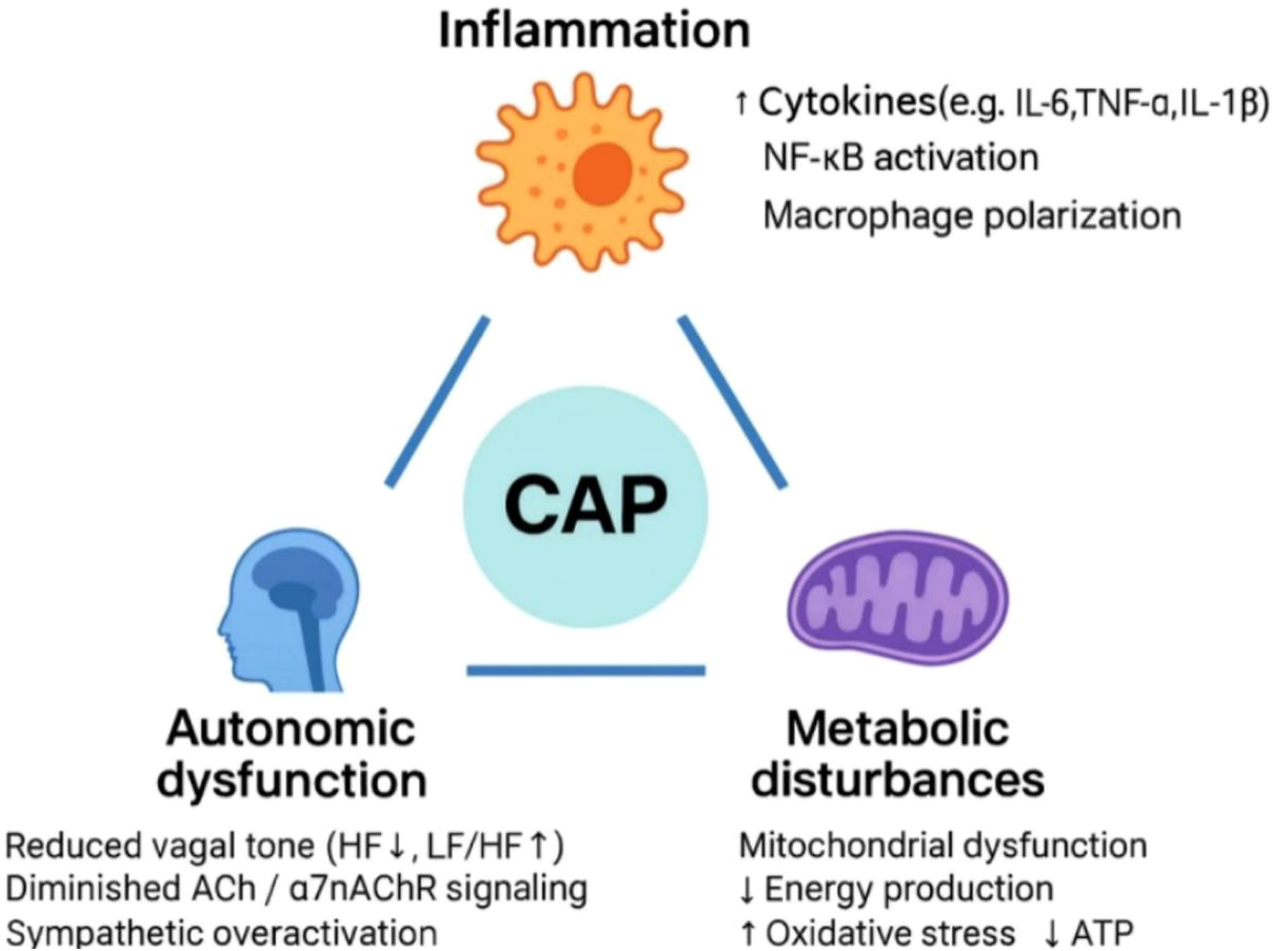
Integrated inflammation–autonomic–metabolic disturbance network and central role of CAP in long COVID. This diagram illustrates the core pathophysiological triad of long COVID—persistent inflammation, autonomic dysfunction, and metabolic disorder—along with the integrative regulatory role of the CAP. Inflammatory features include elevated cytokines, NF-κB activation, and pro-inflammatory macrophage polarization. Autonomic dysfunction is reflected by reduced vagal tone, impaired ACh/α7nAChR signaling, and sympathetic dominance. Metabolic dysregulation encompasses mitochondrial dysfunction, reduced ATP production, and heightened oxidative stress. The connecting lines indicate bidirectional reinforcing interactions among these domains. Positioning CAP at the center highlights its potential role as a neural–immune–metabolic regulatory hub. When CAP-related feedback is weakened, anti-inflammatory regulation may decline, autonomic imbalance may worsen, and metabolic resilience may decrease, collectively contributing to multisystem symptom persistence in long COVID.

We visualize this interconnected pathological triad through a systems-level framework that highlights the bidirectional interactions among inflammation, autonomic imbalance, and metabolic dysfunction, as shown in Figure 1.

## 3 Vagus nerve and the CAP

### 3.1 Anatomy and functions of the vagus nerve

The vagus nerve, as the tenth cranial nerve, serves as the primary parasympathetic pathway in the human body. It innervates numerous visceral organs including the heart, lungs, gastrointestinal tract, liver, and spleen, acting as a communication link between the central nervous system and peripheral organs (Ottaviani and Macefield, 2022). Anatomical studies indicate that approximately 80% of vagus nerve fibers are afferent, while 20% are efferent (Butt et al., 2020). Afferences transmit sensory information from peripheral organs to the NTS in the medulla, where signals undergo central integration. Efferent signals generated from the DMV and nucleus ambiguus subsequently regulate cardiovascular, respiratory, gastrointestinal, and immune functions. This bidirectional neural architecture collectively constitutes the brain-visceral axis, establishing the vagus nerve as a central hub for neuroimmunoregulatory homeostasis (Prescott and Liberles, 2022).

ACh is the primary neurotransmitter of the vagus nerve, mediating its regulatory effects through multiple signaling layers. Vagus efferent fibers can reduce heart rate, enhance gastrointestinal motility, and regulate insulin secretion, while vagus afferent fibers detect peripheral inflammatory signals and transmit them to the brainstem (da Silva et al., 2025; Kawana et al., 2024). Of particular significance is the vagus-immune reflex arc, also known as the CAP (Li L. et al., 2025). When inflammatory signals reach the NTS via vagal afferents, the brainstem generates a reflexive output through the DMV and nucleus ambiguus (Huerta et al., 2025). This signal ultimately triggers the release of ACh, which acts on α7nAChR on the surface of macrophages in organs such as the spleen and liver, thereby inhibiting NF-κB activation and the transcription of proinflammatory cytokines (Andersson and Tracey, 2024; McAllen et al., 2022; Ziegler et al., 2025).

This reflex arc comprises three key steps:

(1) Transmission of peripheral inflammatory signals to the central nervous system via vagal afferent fibers (Jin et al., 2024);
(2) Integration and efferent transmission of signals through the medullary nucleus-dorsal medullary region circuit (Simon et al., 2023);
(3) ACh inhibition of inflammatory responses in peripheral immune cells (Kelly et al., 2022).

When vagus nerve function is impaired, transmission along the CAP reflex arc is disrupted, leading to sustained cytokine release and chronic, unresolved inflammation. Long COVID patients often exhibit reduced HRV, decreased ACh levels, and diminished α7 nAChR expression (Leitzke et al., 2025; Li D. et al., 2023; Marques et al., 2023), suggesting that diminished CAP sensitivity may be one factor contributing to persistent inflammation.

In summary, the vagus nerve-central anti-inflammatory pathway (CAP) serves not only as a crucial anti-inflammatory reflex mechanism but also as a central regulatory hub for maintaining neuro-immune-metabolic balance. This provides an important physiological basis for understanding the systemic regulatory effects of EA.

**FIGURE 2:**
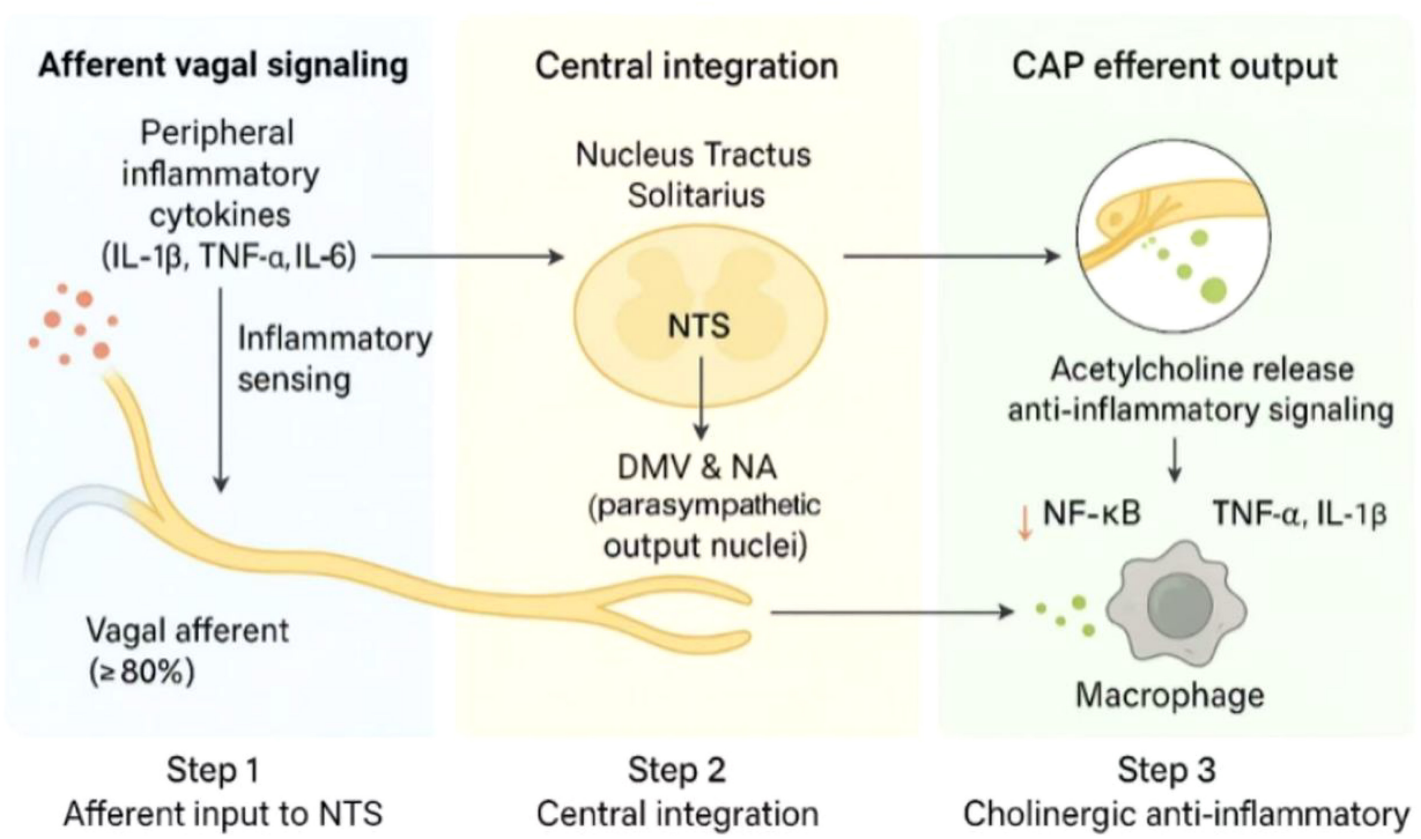
Vagal–CAP reflex arc: afferent sensing, central integration, and efferent anti-inflammatory output. This figure illustrates the three-step structural framework of the vagus nerve CAP. Peripheral inflammatory cytokines (IL-1β, TNF-α, IL-6) activate vagal afferent sensory fibers ( ≥ 80% of vagus fibers), transmitting inflammatory signals to the NTS, where inputs are centrally integrated and relayed via the DMV and the nucleus ambiguus (NA). The vagus nerve efferent fibers subsequently release ACh, which acts on macrophages to inhibit NF-κB activation and reduce the production of proinflammatory cytokines such as TNF-α and IL-1β, constituting the efferent branch of the CAP.

To elucidate how vagal pathways interact with peripheral immune activity, Figure 2 outlines the core structure of the cholinergic anti-inflammatory reflex.

### 3.2 Molecular mechanisms underlying the cholinergic anti-inflammatory pathway

The CAP is fundamentally driven by the activation of α7-nicotinic α7nAChR mediated by ACh (Keever et al., 2024). Multiple downstream signaling cascades converge to regulate inflammatory responses, oxidative stress, and cellular energy metabolism (ElNebrisi et al., 2025).

A key signaling pathway involves the α7nAChR–Janus kinase 2 (JAK2)–signal transducer and activator of transcription 3 (STAT3) axis, which ultimately inhibits NF-κB transcription (Kanauchi et al., 2022). Upon binding of ACh to α7nAChRs, JAK2 is activated and phosphorylates STAT3, promoting its translocation to the cell nucleus while initiating anti-inflammatory gene transcription (ElNebrisi et al., 2025). Activated STAT3 also upregulates cytokine-signaling inhibitor 3 (SOCS3), which exerts negative feedback on upstream inflammatory signals, further suppressing cytokine production (Nakamura et al., 2023). Additionally, the α7nAChR signaling pathway alleviates excessive inflammatory responses by inhibiting NLRP3 inflammasome assembly and reducing IL-1β release (Simon et al., 2023).

CAP activation also initiates the nuclear factor erythroid 2-related factor 2 (Nrf2)-dependent antioxidant pathway, thereby regulating oxidative stress (Bigler et al., 2022). Stimulation of α7nAChRs promotes Nrf2 nuclear translocation, thereby inducing the expression of heme oxygenase-1 (HO-1), superoxide dismutase (SOD), and glutathione peroxidase (GSH-Px) (Zhang Q. et al., 2023). Through the synergistic action of the NF-κB and Nrf2 pathways, CAP simultaneously inhibits inflammatory signaling and enhances antioxidant defense capabilities.

The α7nAChR signaling pathway exhibits cross-regulation with the AMP-activated protein kinase (AMPK)-SIRT1 metabolic axis. ACh can indirectly enhance AMPK phosphorylation through calcium-dependent mechanisms (Lovy et al., 2020). Beyond anti-inflammatory and antioxidant effects, ACh modulates cellular energy metabolism through the AMPK-SIRT1-PGC-1α cascade (Keever et al., 2024). AMPK activation enhances energy utilization efficiency and suppresses inflammatory responses, while SIRT1 promotes PGC-1α activity via deacetylation, thereby boosting mitochondrial biogenesis and antioxidant capacity (Abu Shelbayeh et al., 2023; Hao et al., 2025).

Collectively, these mechanisms demonstrate that CAP functions not only as a neural reflex arc but also as an integrative hub linking neuromodulation, immune homeostasis, and metabolic remodeling.

**FIGURE 3:**
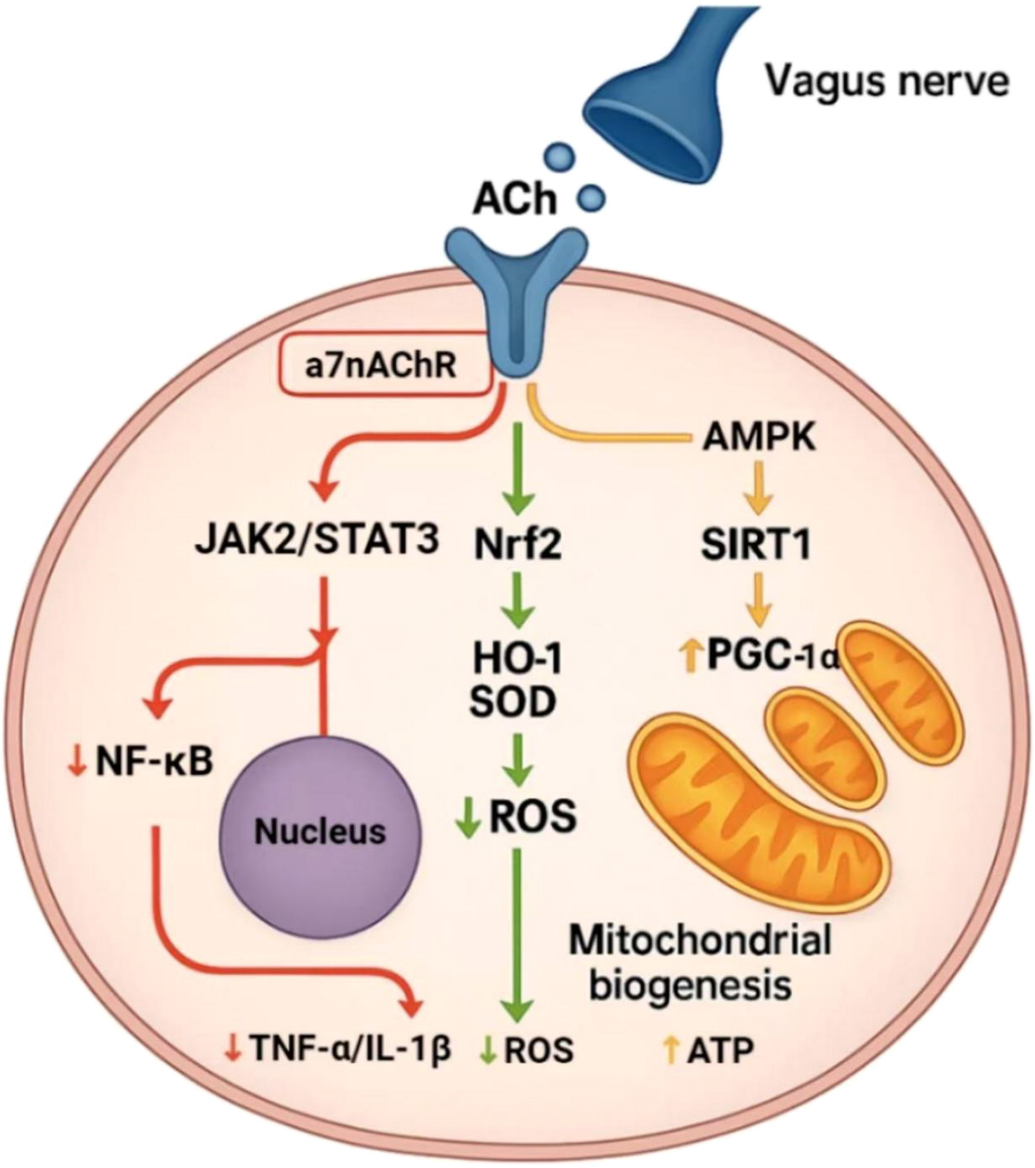
α7nAChR-mediated anti-inflammatory, antioxidant, and metabolic signaling pathways. This figure illustrates the intracellular signaling pathways activated by ACh binding to the α7nAChR. Engagement of α7nAChR triggers the JAK2/STAT3 cascade, which suppresses NF-κB activity and reduces downstream pro-inflammatory cytokines such as TNF-α and IL-1β. In parallel, α7nAChR signaling promotes Nrf2 activation, leading to upregulation of antioxidant enzymes including HO-1 and SOD and a consequent reduction in reactive oxygen species (ROS). Additionally, cholinergic signaling interacts with the AMPK–SIRT1–PGC-1α metabolic axis to enhance mitochondrial biogenesis and increase ATP production. Together, these converging mechanisms represent the core anti-inflammatory, antioxidant, and metabolic effects of the CAP.

Therefore, the downstream molecular pathways linking α7 nAChR activation to inflammation control and metabolic recovery are shown in Figure 3.

### 3.3 Impaired vagal–cholinergic anti-inflammatory signaling in COVID-19 and long COVID

Emerging evidence suggests that COVID-19 and long COVID may involve disturbances in vagal activity, autonomic balance, and cholinergic anti-inflammatory regulation. During acute SARS-CoV-2 infection, systemic inflammation and cytokine overactivation may affect both central and peripheral components of the nervous system, including brainstem autonomic circuits and vagal regulatory pathways (Hajiasgharzadeh et al., 2022; Zubair et al., 2020). In the post-acute phase, subsets of patients with long COVID show persistent autonomic abnormalities, including reduced heart rate variability (HRV), sympathetic predominance, orthostatic intolerance, and fatigue-related physiological instability (Menezes Junior et al., 2022; Qin et al., 2025). These findings suggest that impaired vagal regulation may contribute to persistent inflammatory and metabolic disturbances in long COVID.

In this review, we use the term CAP hyporesponsiveness as a provisional mechanistic descriptor rather than as a validated clinical diagnosis. This term refers to a pattern in which reduced vagal activity, insufficient cholinergic anti-inflammatory signaling, persistent inflammatory activation, and metabolic–mitochondrial disturbance may co-occur. It is intended to organize convergent observations across autonomic, immune, and metabolic studies, rather than to define a new disease entity or a formally established syndrome.

This distinction is important because CAP hyporesponsiveness should not be equated with general post-COVID autonomic dysfunction. General autonomic dysfunction is usually reflected by reduced HRV, sympathetic predominance, orthostatic intolerance, POTS-like symptoms, palpitations, sleep disturbance, or anxiety-related autonomic instability. By contrast, a CAP hyporesponsiveness pattern would require evidence beyond autonomic symptoms or HRV abnormalities. It would involve candidate indicators of impaired cholinergic anti-inflammatory feedback, including altered ACh/ChE balance, reduced or dysregulated α7nAChR-related signaling, persistent activation of inflammatory pathways such as NF-κB or NLRP3, elevated inflammatory markers, and metabolic or oxidative stress abnormalities (Bellocchi et al., 2022; Lage et al., 2025; Mehranfard and Speth, 2022; Oka et al., 2023; Yin et al., 2024).

Accordingly, the domains summarized in Table 1 should be interpreted as candidate mechanistic domains, not as diagnostic criteria. At present, no validated clinical threshold exists for defining CAP hyporesponsiveness in long COVID. HRV reduction alone is insufficient to establish CAP impairment, because HRV is an indirect marker of autonomic regulation and is not specific to cholinergic anti-inflammatory signaling. Similarly, inflammatory markers such as IL-6, TNF-α, IL-1β, or CRP may indicate persistent immune activation but cannot independently identify CAP dysfunction. Therefore, future studies should test whether combined autonomic, cholinergic, inflammatory, and metabolic markers can reproducibly identify a subgroup of patients with long COVID who show impaired vagal–cholinergic anti-inflammatory regulation.

**TABLE 1:** Provisional mechanistic domains and candidate markers of CAP hyporesponsiveness in long COVID.

Mechanistic domain	Candidate marker/measurement	Physiological meaning	Possible relevance to CAP hyporesponsiveness
Autonomic regulation	HRV-HF ↓; RMSSD ↓; LF/HF ↑; orthostatic intolerance	Reduced parasympathetic tone or sympathovagal imbalance	Suggests reduced vagal regulatory capacity, but is not CAP-specific
Cholinergic signaling	ACh ↓; ChE ↑	Reduced cholinergic availability or accelerated acetylcholine degradation	May indicate weakened cholinergic input to anti-inflammatory regulation
CAP-related inflammatory signaling	α7nAChR ↓; NF-κB ↑; NLRP3 ↑	Impaired cholinergic anti-inflammatory signaling and inflammasome activation	Suggests reduced responsiveness of α7nAChR-mediated immune regulation
Systemic inflammatory burden	IL-6 ↑; TNF-α↑; IL-1β↑; CRP ↑	Persistent low-grade or fluctuating inflammatory activation	May reflect insufficient inflammatory resolution, but is not specific to CAP dysfunction
Metabolic-mitochondrial regulation	AMPK ↓; SIRT1 ↓; PGC-1α↓; ROS ↑; lactate ↑	Impaired bioenergetic regulation, oxidative stress, and mitochondrial dysfunction	Supports possible interaction between inflammatory persistence and metabolic stress
Clinical manifestations	Fatigue, exercise intolerance, heart rate instability, cognitive symptoms	Multisystem symptom expression involving autonomic, immune, and metabolic domains	Compatible with CAP-related dysregulation, but clinically nonspecific

These domains and markers are not proposed as diagnostic criteria. They represent candidate mechanistic indicators that require validation in prospective clinical studies. HRV, heart rate variability; HF, high frequency; RMSSD, root mean square of successive differences; LF, low frequency; ACh, acetylcholine; ChE, cholinesterase; α7nAChR, alpha-7 nicotinic acetylcholine receptor; NF-κB, nuclear factor kappa-B; NLRP3, NOD-like receptor pyrin domain-containing 3; CRP, C-reactive protein; AMPK, AMP-activated protein kinase; SIRT1, sirtuin 1; PGC-1α, peroxisome proliferator-activated receptor gamma coactivator-1α; ROS, reactive oxygen species.

From this perspective, CAP hyporesponsiveness represents a research framework for investigating how impaired vagal anti-inflammatory feedback may contribute to persistent inflammation, metabolic dysfunction, and multisystem symptoms in long COVID. This framework remains hypothesis-generating and requires validation through longitudinal studies, multimodal autonomic assessment, immune and metabolic profiling, and interventional trials targeting vagal or cholinergic regulatory pathways.

### 3.4 Summary: multilayer integration of CAP and its impact on EA

The vagus nerve-CAP axis serves as a core regulatory system for maintaining immune homeostasis, and its dysfunction appears to directly contribute to the chronic inflammation and systemic fatigue reported in long COVID. We propose that CAP functions not merely as an anti-inflammatory reflex, but as a pivotal nexus where neural, immune, and metabolic regulation converge. Rather than operating through a single pathway, CAP influences several levels of signaling. It modulates the α7nAChR–JAK2–STAT3–NF-κB axis, which curbs inflammatory activity, while also engaging the AMPK–SIRT1–PGC-1α pathway that supports mitochondrial function and broader metabolic stability. In our interpretation, this combination allows CAP to participate in a neuro–immune–metabolic network in which neural signals help shape both immune responses and cellular energy management.

Based on current findings, we think the vagus–CAP system may coordinate multilayer integration partly through AMPK–SIRT1–PGC-1α signaling. This line of reasoning provides the starting point for the integrated EA–CAP–AMPK–SIRT1–PGC-1α model that we develop in the next section.

## 4 Evidence for EA-induced activation of the vagus–CAP axis

### 4.1 Evidence from animal studies

Animal studies provide the main preclinical basis for linking EA to vagal–cholinergic anti-inflammatory regulation. Existing evidence suggests that EA can modulate systemic inflammation through somatic–autonomic reflex mechanisms, with CAP-related signaling representing one important pathway (Li et al., 2021; Wang et al., 2025). However, the strength of evidence differs across studies; therefore, the animal evidence is summarized in Table 2 according to model, acupoint and stimulation parameters, outcomes, mechanistic indicators, and evidence level.

**TABLE 2:** Representative animal evidence for EA modulation of vagal-cholinergic anti-inflammatory and metabolic pathways.

Study model	Acupoint	Stimulation parameters	Main outcomes	CAP-related mechanistic evidence	Evidence level
LPS-induced endotoxemia/septic mice (Lv et al., 2022)	ST36	EA at ST36; 0.1 mA, continuous wave, 10 Hz; 30 min/day; once daily for 3 days before LPS challenge.	Improved survival, symptom score, ear temperature, pulmonary and intestinal injury; blunted cytokine storm and reduced T-cell apoptosis/pyroptosis.	Systemic anti-inflammatory effect required T cells, but vagus nerve or alpha7nAChR dependence was not directly tested.	Indirect anti-inflammatory animal evidence; not CAP-specific
LPS-induced sepsis/intestinal inflammation and flora dysbiosis (Zhan et al., 2022)	Bilateral ST36	Needles inserted 3 mm into bilateral ST36; dense wave, 2.5 mA, 2-100 Hz; 30 min/day for 5 days before LPS injection.	Reduced IL-1beta, IL-6 and TNF-alpha; increased IL-10; reduced LDH release and apoptosis; improved intestinal flora diversity.	Downregulated TLR4, MyD88 and NF-kappaB signaling; CAP dependence was not directly tested.	Indirect anti-inflammatory animal evidence; CAP not directly tested
CLP-induced sepsis lung injury (Shi et al., 2022)	ST36	EA at ST36; 1 mA, 2 Hz dense wave; 30 min/day for 5 consecutive days before CLP.	Improved survival; reduced lung edema and pathological injury; decreased TNF-alpha and IL-1beta; increased IL-10.	EA restored alpha7nAChR expression and inhibited NF-kappaB. The alpha7nAChR antagonist MLA attenuated EA effects; GTS-21 was used as an alpha7nAChR agonist comparator.	Direct alpha7nAChR-related animal evidence
Postoperative ileus/intestinal inflammation (Yang et al., 2021)	Bilateral ST36	Two stainless steel needles inserted 3 mm into bilateral ST36; 1 mA, 10 Hz, pulse width 0.4 ms; 20 min.	Promoted gastrointestinal motility and reduced intestinal inflammation; decreased inflammatory cytokine production in intestinal muscularis.	EA activated alpha7nAChR-mediated JAK2/STAT3 signaling in macrophages. Effects were tested with alpha7nAChR antagonists, JAK2/STAT3 inhibitors, cervical vagotomy and sub-diaphragmatic vagotomy.	Direct vagal-CAP-related animal evidence
Acute pancreatitis induced by caerulein or pancreatic duct ligation (Zhang L. et al., 2021)	Bilateral ST36	Needles inserted about 3 mm into bilateral ST36; 2 mA, 2/15 Hz; 20 min. Applied immediately after first caerulein injection or 30 min and 24 h after PDL operation.	Increased vagus nerve activity; reduced systemic inflammation; alleviated pancreatic histopathology and leukocyte infiltration.	EA increased alpha7nAChR-positive macrophages. Vagotomy and MLA attenuated anti-inflammatory and pancreatic protective effects.	Direct vagus/CAP-related animal evidence
Dry eye disease rabbit model induced by scopolamine hydrobromide (Ding et al., 2021)	Periocular acupoints: BL1, BL2, SJ23, EX-HN5 and GB1	EA once daily for 14 consecutive days; 15 min/session; dense-disperse wave 4/20 Hz; pulse width 0.5 ms; 1 mA.	Improved corneal epithelial injury, tear secretion, lacrimal gland pathology and inflammatory cytokines including IL-1, MIP-1beta, TNF-alpha and IL-8.	EA upregulated alpha7nAChR and downregulated NF-kappaB. The alpha7nAChR antagonist alpha-BGT reversed EA effects.	Direct alpha7nAChR/ NF-kappaB-related animal evidence; not vagus-specific
LPS-induced ALI/ARDS mice (Zhang et al., 2022)	Bilateral ST36	EA at bilateral ST36; 0.5 mA, 4/20 Hz; 20 min/day for 3 consecutive days after LPS challenge.	Reduced alveolar inflammatory cell/protein exudation, lung pathological injury, IL-1beta and TNF-alpha expression, ferroptosis markers and mortality.	EA activated alpha7nAChR in lung tissue mainly through sciatic nerve and cervical vagus nerve pathways. Effects were eliminated by MLA and reversed by sciatic nerve transection, cervical vagotomy or erastin.	Direct alpha7nAChR/ vagus-related pulmonary evidence
HFD-induced MAFLD with intestinal barrier disruption (Wang et al., 2025)	ST36	EA at ST36; 45 min/session, 2 mA, 50 Hz; alternate days for 4 weeks.	Reduced body weight, liver weight, visceral fat index, liver injury, dyslipidemia, hepatic steatosis, fibrosis and inflammation; reduced gut permeability and intestinal epithelial disruption; upregulated tight-junction proteins.	EA enriched alpha7nAChR and HO-1 expression and reduced p38 MAPK/NF-kappaB activation. Protective effects were abolished by alpha-BGT or intestine-specific HO-1 deletion.	Direct/partial CAP-related intestinal barrier evidence
T2DM rat model/skeletal muscle glycolipid metabolism (Luo et al., 2025)	ST36, SP6 and EX-B3	EA at ST36, SP6 and EX-B3; continuous wave, 15 Hz; 20 min/day, 6 days/week for 4 weeks.	Reduced random and fasting blood glucose, body weight, TG, LDL-C, FINS, C-peptide and HOMA-IR; improved insulin sensitivity index; increased skeletal muscle ATP.	EA increased AMPK, PGC-1alpha, TFAM and GLUT4 expression at protein and mRNA levels; effects were abolished by AMPK inhibitor Compound C.	Metabolic-axis animal evidence; not CAP-specific

Direct CAP-related animal evidence refers to studies using pathway-disruption approaches such as vagotomy, alpha7nAChR antagonism, alpha-BGT/MLA, or related cholinergic pathway manipulation. Indirect anti-inflammatory evidence refers to EA studies showing anti-inflammatory effects without direct testing of vagal or alpha7nAChR dependence. Metabolic-axis studies are included to support the AMPK/PGC-1alpha component of the integrated model and should not be interpreted as direct evidence for CAP activation. EA, electroacupuncture; CAP, cholinergic anti-inflammatory pathway; alpha7nAChR, alpha-7 nicotinic acetylcholine receptor; ST36, Zusanli; SP6, Sanyinjiao; EX-B3, Weiwanxiashu; BL1, Jingming; BL2, Cuanzhu; SJ23, Sizhukong; EX-HN5, Taiyang; GB1, Tongziliao; LPS, lipopolysaccharide; CLP, cecal ligation and puncture; ALI/ARDS, acute lung injury/acute respiratory distress syndrome; MAFLD, metabolic dysfunction-associated fatty liver disease; T2DM, type 2 diabetes mellitus; MLA, methyllycaconitine; alpha-BGT, alpha-bungarotoxin; HFD, high-fat diet; HO-1, heme oxygenase-1; MAPK, mitogen-activated protein kinase; NF-kappaB, nuclear factor-kappa B; PDL, pancreatic duct ligation; FINS, fasting insulin; HOMA-IR, homeostasis model assessment of insulin resistance; TG, triglycerides; LDL-C, low-density lipoprotein cholesterol.

In LPS-induced endotoxemia and sepsis-related models, EA at ST36 reduced inflammatory cytokine production, improved survival-related outcomes, attenuated tissue injury, and modulated immune responses (Lv et al., 2022; Zhan et al., 2022). These findings support anti-inflammatory effects of EA, but they remain indirect when vagotomy or α7nAChR blockade was not used. Stronger mechanistic evidence comes from sepsis-related lung injury and postoperative ileus models, where EA restored α7nAChR expression, inhibited NF-κB signaling, activated α7nAChR-mediated JAK2/STAT3 signaling, and showed reduced effects after α7nAChR antagonism or vagotomy (Shi et al., 2022; Yang et al., 2021).

Organ-specific inflammatory models further support vagal–cholinergic involvement. In acute pancreatitis, EA at ST36 increased vagal activity, reduced pancreatic inflammation, increased α7nAChR-positive macrophages, and its protective effects were weakened by vagotomy or methyllycaconitine (Zhang L. et al., 2021). In dry eye disease, EA reduced lacrimal gland inflammation, upregulated α7nAChR, and inhibited NF-κB, although vagal dependence was not directly tested (Ding et al., 2021). Recent animal studies also support EA effects on pulmonary inflammation, intestinal barrier disruption, and AMPK/PGC-1α-related metabolic regulation (Luo et al., 2025; Wang et al., 2025; Zhang et al., 2022). Overall, the strongest animal evidence comes from studies using vagotomy, α7nAChR antagonists, or related pathway-disruption approaches (Shi et al., 2022; Yang et al., 2021; Zhang L. et al., 2021), whereas other findings should be interpreted as indirect anti-inflammatory or metabolic support for the proposed framework.

### 4.2 Evidence from clinical studies

Clinical studies provide supportive evidence for the modulatory effects of electroacupuncture on autonomic nervous system function, inflammation, and neurophysiological function, although this evidence is primarily indirect (Armstrong et al., 2020; Gagliardi et al., 2023; Lee et al., 2011; Li et al., 2022; Yang S. et al., 2024; Zhou et al., 2024). Unlike animal studies, human research rarely directly tests vagus nerve-CAP dependence using pathway disruption methods. Therefore, clinical evidence is best interpreted through measurable indicators such as HRV, circulating inflammatory markers, symptom changes, and neuroimaging results (Armstrong et al., 2020; Gagliardi et al., 2023; Huang et al., 2022; Lee et al., 2011; Peihong et al., 2021).

Table 3 summarizes representative clinical studies according to study population or disease condition, acupoints, stimulation parameters, study results, physiological or mechanistic indicators, and level of evidence. In both healthy volunteers and patients with inflammation-related diseases, reports have indicated that electroacupuncture can influence autonomic balance, inflammatory markers, pain or gastrointestinal symptoms, and central regulatory networks (Armstrong et al., 2020; Gagliardi et al., 2023; Hao et al., 2023; Huang et al., 2022; Jia et al., 2011; Lee et al., 2011; Shi et al., 2020). These findings are consistent with autonomic and neuroimmune modulation associated with electroacupuncture; however, they should not be interpreted as direct evidence of CAP activation unless markers of cholinergic or inflammatory pathways are simultaneously measured.

**TABLE 3:** Representative clinical evidence supporting EA-induced activation of the vagus–CAP pathway.

Population/condition	Acupoint	Stimulation parameters	Clinical outcomes	Physiological/mechanistic indicators	Evidence level
Healthy adults/HRV frequency-response study (Jia et al., 2011)	Bilateral ST36 and ST37	Sham, 2 Hz, 15 Hz, and 50 Hz EA sessions; intensity adjusted to individual tolerance.	No disease outcome; autonomic response assessed across stimulation frequencies.	HRV indices changed in a frequency-dependent manner; supports EA-related autonomic modulation but does not test inflammatory or cholinergic markers.	Autonomic physiological evidence; indirect CAP relevance
Healthy volunteers/HRV randomized clinical trial (Jia et al., 2011)	LI4 and LI11	2 Hz or 120 Hz EA; 15 min; randomized crossover design.	No disease outcome; acute autonomic response assessed before and after EA.	120 Hz EA increased SDNN, whereas HF, LF and LF/HF did not differ significantly between low- and high-frequency EA.	Autonomic physiological evidence; indirect CAP relevance
Healthy or stress-related participants receiving Battlefield Acupuncture protocol (Armstrong et al., 2020)	Five auricular BFA points	Low-frequency EA 2.5 Hz or mid-frequency EA 15 Hz; single treatment session.	Low-frequency EA reduced sympathetic stress and improved autonomic recovery.	2.5 Hz EA improved HRV total power, reduced sympathetic stress index and increased HF vagal tone.	Autonomic physiological evidence; indirect CAP relevance
Healthy subjects/auricular electroacupuncture and microcirculation (Gagliardi et al., 2023)	Auricular stimulation points	Low- and high-frequency auricular EA; crossover design.	Low-frequency stimulation increased cutaneous microcirculatory flux without significant changes in blood pressure or heart rate.	Findings suggest autonomic-vascular regulation, but inflammatory or cholinergic markers were not measured.	Autonomic-vascular physiological evidence
Knee osteoarthritis patients/inflammatory-marker clinical study (Shi et al., 2020)	Knee local acupoints and distal points used in the ATKOA protocol	EA or manual acupuncture; 24 sessions over 8 weeks in the parent ATKOA clinical trial.	Improved pain and function outcomes measured by VAS and WOMAC.	EA and manual acupuncture reduced TNF-α and IL-1β and increased IL-13; TNF-α reduction was greater with EA than MA.	Inflammatory-marker clinical evidence; indirect CAP relevance
Ulcerative colitis patients/meta-analysis and acupoint selection study (Hao et al., 2023)	Commonly selected acupoints varied across trials	EA protocols varied across included studies.	Improved clinical efficacy and disease-related outcomes across included UC trials.	Supports potential gut-autonomic-immune regulation, but most trials did not directly measure vagal-CAP markers.	Clinical inflammatory-disease evidence; indirect CAP relevance
Healthy subjects/ST36 task-based fMRI synthesis (Huang et al., 2022)	ST36	Acupuncture at ST36; stimulation protocols varied across included fMRI studies.	No direct therapeutic outcome; central response to ST36 stimulation assessed.	ST36 stimulation modulated brain regions involved in somatosensory, limbic and autonomic-related processing.	Neuroimaging evidence; indirect autonomic relevance

Clinical evidence was graded according to whether studies measured autonomic indicators, inflammatory markers, neuroimaging changes, or symptom outcomes. Because most human studies did not directly test vagal-CAP dependence or α7nAChR-mediated signaling, these findings should be interpreted as indirect clinical support rather than definitive evidence of CAP activation. HRV, heart rate variability; HF, high frequency; LF, low frequency; SDNN, standard deviation of normal-to-normal intervals; BFA, Battlefield Acupuncture; VAS, visual analogue scale; WOMAC, Western Ontario and McMaster Universities Osteoarthritis Index; UC, ulcerative colitis; EA, electroacupuncture; CAP, cholinergic anti-inflammatory pathway.

Overall, current clinical evidence supports the view that electroacupuncture (EA) may influence vagus nerve-related physiological regulatory mechanisms in the human body (Armstrong et al., 2020; Gagliardi et al., 2023; Lee et al., 2011; Li et al., 2022; Yang S. et al., 2024; Zhou et al., 2024). However, since most studies rely on heart rate variability (HRV), inflammatory markers, neuroimaging changes, or symptomatic outcomes rather than direct detection of α7nAChR-mediated CAP signaling, these findings should be regarded as supportive evidence of electroacupuncture-induced CAP activation rather than conclusive evidence (Hao et al., 2023; Huang et al., 2022; Lee et al., 2011; Peihong et al., 2021; Sun et al., 2023).

**FIGURE 4:**
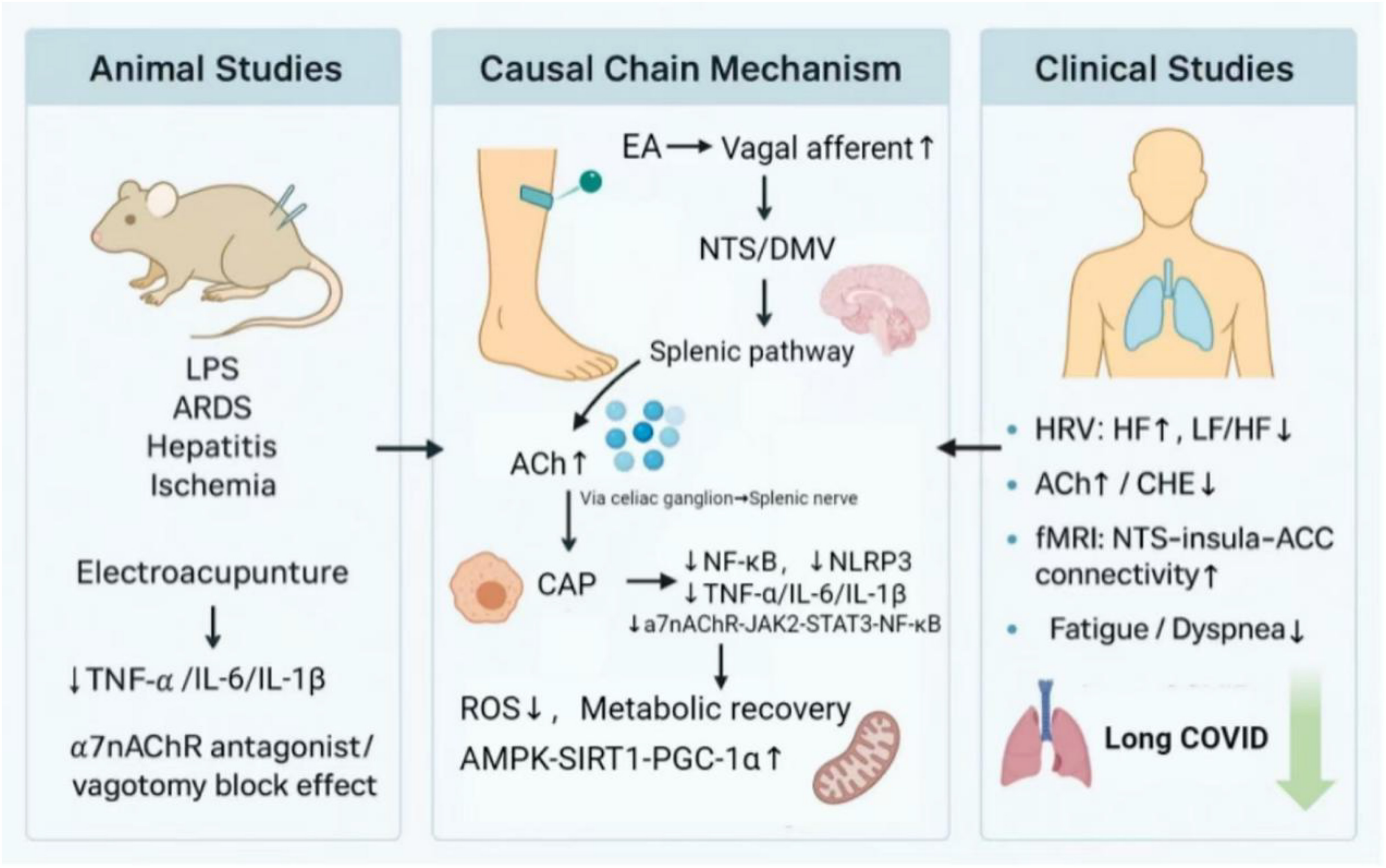
Evidence chain linking EA, CAP activation, and clinical outcomes. This figure summarizes converging evidence supporting the mechanism of EA: it activates the vagus nerve afferent pathway, initiates the CAP, and subsequently promotes downstream immunometabolic regulation. Animal model studies (including LPS-induced inflammation, acute respiratory distress syndrome, ischemia, and hepatitis models) demonstrate that EA reduces proinflammatory cytokine levels. This effect is eliminated by vagus nerve transection or α7nAChR blockade, indicating CAP pathway dependence. Mechanistic studies reveal that vagus nerve input to the nucleus accumbens/medullary vagus nucleus complex enhances ACh signaling and α7nAChR activation, thereby inhibiting the NF-κB/NLRP3 pathway and reducing TNF-α, IL-6, and IL-1β levels. CAP activation also improves redox balance and mitochondrial function through synergistic interaction with the AMPK-SIRT1-PGC-1α signaling pathway. Clinical and related neuromodulation studies suggest changes in HRV, cholinergic indicators, neurophysiological regulation, and symptom-related outcomes; however, direct evidence in long COVID remains limited and requires further validation. These findings collectively form a coherent cross-evidence framework, while the full causal sequence in long COVID remains to be validated.

Based on findings from animal models and clinical studies, we propose an evidence-based approach that links electroacupuncture stimulation, vagal-cholinergic modulation, inflammation regulation, and downstream metabolic effects (Armstrong et al., 2020; Gagliardi et al., 2023; Hao et al., 2023; Huang et al., 2022; Lee et al., 2011; Li et al., 2021; Lv et al., 2022; Peihong et al., 2021; Shi et al., 2022; Sun et al., 2023; Yang et al., 2021; Zhan et al., 2022; Zhang L. et al., 2021; Zhang et al., 2022). This integrated framework is illustrated in Figure 4.

### 4.3 Mechanistic convergence: from neural signaling to molecular effects

Animal and clinical studies reveal remarkably similar mechanisms. At the neural level, EA enhances vagal excitability, manifested as increased HF HRV components and heightened reflex tachycardia activity (Armstrong et al., 2020; Gagliardi et al., 2023). At the molecular level, EA upregulates α7nAChR, AMPK, and SIRT1 expression while inhibiting NF-κB pathway and NLRP3 inflammasome activation (Chen Y. et al., 2024; Zhang et al., 2022). The synergistic interaction between neural and molecular effects indicates that EA simultaneously restores reflexive autonomic regulation and reestablishes intracellular signaling pathways, thereby achieving dual reconstruction of inflammatory and metabolic equilibrium.

Available evidence indicates that transcutaneous electrical stimulation activation of the CAP reflex not only suppresses proinflammatory responses but also improves mitochondrial metabolism and energy supply through the AMPK-SIRT1-PGC-1α pathway. These multilayered effects mirror the integrative neuro–immune–metabolic mechanisms described in section 3 and provide empirical support for the mechanistic model developed in the subsequent sections.

### 4.4 Mechanistic evidence chain supported by current research

Existing animal and clinical studies support a layered mechanistic evidence chain through which EA may influence peripheral sensory input, central autonomic regulation, immune signaling, and metabolic responses. Experimental evidence shows that EA stimulation at points such as ST36 and PC6 enhances somatosensory input along the skin–spinal–brainstem pathway, increasing vagal afferent activity, as reflected by elevated HF components of HRV and enhanced firing of vagal sensory fibers (He et al., 2025; Li et al., 2022). Blocking vagal afferents attenuates EA’s anti-inflammatory effects, indicating that enhanced vagal input represents an initiating step in the mechanism (Benghanem et al., 2022; Fan, 2023). After integration within the NTS and DMV, strengthened afferent signals activate the CAP and promote ACh release; α7nAChR antagonists markedly disrupt this process, underscoring the indispensable role of CAP (Yang L. et al., 2024; Zhang et al., 2025).

At the level of the peripheral immune system, substantial evidence indicates that EA can upregulate α7nAChR, inhibit NF-κB and NLRP3 inflammasome activation, and reduce the production of inflammatory cytokines such as TNF-α, IL-6, and IL-1β (Guo L. et al., 2024; Zhang et al., 2022). These findings suggest that acupuncture exerts systemic anti-inflammatory effects through the CAP-α7nAChR-NF-κB/NLRP3 axis. In parallel, EA activates the AMPK–SIRT1–PGC-1α signaling cascade, promoting mitochondrial biogenesis and oxidative metabolism, reducing ROS accumulation, and stabilizing mitochondrial membrane potential—collectively alleviating inflammation–metabolism coupling dysfunction (Guo L. et al., 2024; Yuan et al., 2025). Clinical studies further indicate that long COVID patients receiving EA treatment demonstrated improvements in fatigue (Kim S. et al., 2023), dyspnea (Joshi and Biswal, 2022), abnormal heart rate (Liu F. et al., 2024), and low energy levels. Concurrently, they exhibited reduced inflammatory marker levels and enhanced HRV (Kim L. et al., 2023).

Based on existing evidence, a coherent causal framework can be constructed: peripheral EA stimulation enhances the transmission of vagal afferent signals; central integration activates the CAP; downstream pathways suppress inflammatory cascades and restore mitochondrial metabolism; these changes ultimately promote the recovery of systemic homeostasis.

### 4.5 Summary

In summary, current evidence supports a layered interpretation of vagal-cholinergic and metabolic regulation associated with electroacupuncture. The strongest evidence comes from animal studies, in which vagotomy, α7nAChR antagonism, or blockade of related pathways attenuated the effects of EA, supporting the involvement of vagal-CAP-related signaling in selected inflammatory models (Shi et al., 2022; Yang et al., 2021; Zhang L. et al., 2021). Other animal and clinical studies have demonstrated alterations in α7nAChR/NF-κB signaling, inflammatory markers, HRV, neuroimaging responses, or symptomatic outcomes, which are consistent with autonomic or neuroimmune modulation but do not directly prove CAP activation (Armstrong et al., 2020; Ding et al., 2021; Gagliardi et al., 2023; Hao et al., 2023; Huang et al., 2022; Lee et al., 2011; Li et al., 2022; Lv et al., 2022; Peihong et al., 2021; Sun et al., 2023; Yang S. et al., 2024; Zhan et al., 2022; Zhang et al., 2022; Zhou et al., 2024).

Evidence linking EA to AMPK–SIRT1–PGC-1α-related metabolic regulation remains supportive rather than definitive for CAP activation (Luo et al., 2025). Thus, the EA–CAP–AMPK–SIRT1–PGC-1α framework developed in the following section should be presented as an evidence-informed mechanistic model integrating direct preclinical evidence, indirect clinical indicators, and hypothesis-generating inference, not as a fully validated causal pathway.

## 5 The EA–CAP–AMPK–SIRT1–PGC-1α integrated mechanistic model

Building upon evidence from animal experiments, clinical studies, and neurophysiological research, this section develops three interpretive components of the proposed framework. First, the proposed EA–CAP–AMPK–SIRT1–PGC-1α integrated mechanistic model explains how EA coordinates anti-inflammatory activity with metabolic reconstruction through dual converging pathways. Second, we introduce the NRM hypothesis to explain how EA promotes central rhythmic synchronization. Finally, we employ the NIM loop framework to elucidate the mechanism by which EA facilitates multisystem homeostasis restoration through a hierarchical process.

### 5.1 Systemic regulatory features of EA

Previous animal and clinical studies have revealed the effects of electrically stimulated vagus nerve-cardiovascular activation pathways on multiple systems. To fully elucidate these effects, they must be interpreted through a systems physiology perspective. As a form of neuromodulatory intervention, EA exerts effects far beyond local stimulation. Contemporary research indicates that EA activates specific sensory nerve endings and initiates somatic-visceral reflex arcs, achieving bidirectional regulation of autonomic efferent functions. This, in turn, exerts extensive regulatory influences on immune, metabolic, and endocrine networks (Dou et al., 2023; Yang et al., 2022).

Long COVID is a multisystem disorder characterized by persistent inflammation, dysregulation of energy metabolism, and autonomic imbalance. In the context of long COVID, EA may have potential systemic relevance because it can modulate autonomic reflex pathways, inflammatory signaling, and metabolic regulation. By influencing vagal-related reflex activity, EA may contribute to reduced pro-inflammatory signaling and improved autonomic–cardiopulmonary regulation, although direct evidence in long COVID remains limited (Chen Y. et al., 2024). Through the AMPK-SIRT1-PGC-1α axis, EA promotes mitochondrial biogenesis and homeostasis maintenance, reduces reactive oxygen species accumulation, and restores cellular energy metabolism (Landgraaf et al., 2023). At the central nervous level, NRM enhances synchronization between central and peripheral autonomic neural circuits, driving the reorganization of autonomic regulatory pathways (Fernández-Hernando et al., 2023; Rodrigues et al., 2024). These multi-level actions collectively support the synergistic reconstruction of the neuro-immune-metabolic network.

Therefore, EA should not be viewed merely as localized nerve stimulation but rather as a multi-axial neuromodulation system capable of establishing dynamic equilibrium across neural, immune, and metabolic domains. This systems-level perspective provides the theoretical foundation for applying EA to long COVID and other complex chronic diseases characterized by multi-network dysregulation.

Table 4 summarizes the systemic dysregulation underlying major long COVID symptoms and their corresponding EA regulatory mechanisms.

**TABLE 4:** System-level disturbances underlying major long-COVID symptoms and the regulatory actions of EA.

Symptom	System domain	Key mechanisms in long COVID	Potential actions of EA
Fatigue, brain fog, sleep disturbance	Neuro–immune–metabolic network	Chronic low-grade inflammation; reduced vagal tone; impaired mitochondrial oxidative phosphorylation; excess ROS accumulation	Improves autonomic balance and HRV; enhances vagal–CAP signaling; mitigates neuroinflammation; promotes mitochondrial biogenesis and redox homeostasis
Dyspnea	Pulmonary–immune interface	Persistent airway or interstitial inflammation; compromised ventilatory mechanics; dysregulated inflammatory resolution	Modulates vagal–pulmonary reflexes; attenuates airway inflammation; supports recovery of respiratory function
Palpitations/Postural orthostatic tachycardia syndrome (POTS)	Cardiovascular autonomic system	Sympathovagal imbalance; impaired baroreflex sensitivity; orthostatic dysregulation	Rebalances autonomic output; strengthens vagal activity; stabilizes HRV and baroreflex function
Cognitive impairment	Central neuro–immune circuitry	Microglial overactivation; disrupted neuroimmune feedback; reduced CAP responsiveness	Activates the vagal–CAP reflex; suppresses central inflammation; improves connectivity within central autonomic networks

### 5.2 EA–CAP–AMPK–SIRT1–PGC-1α integrated mechanistic model

**FIGURE 5:**
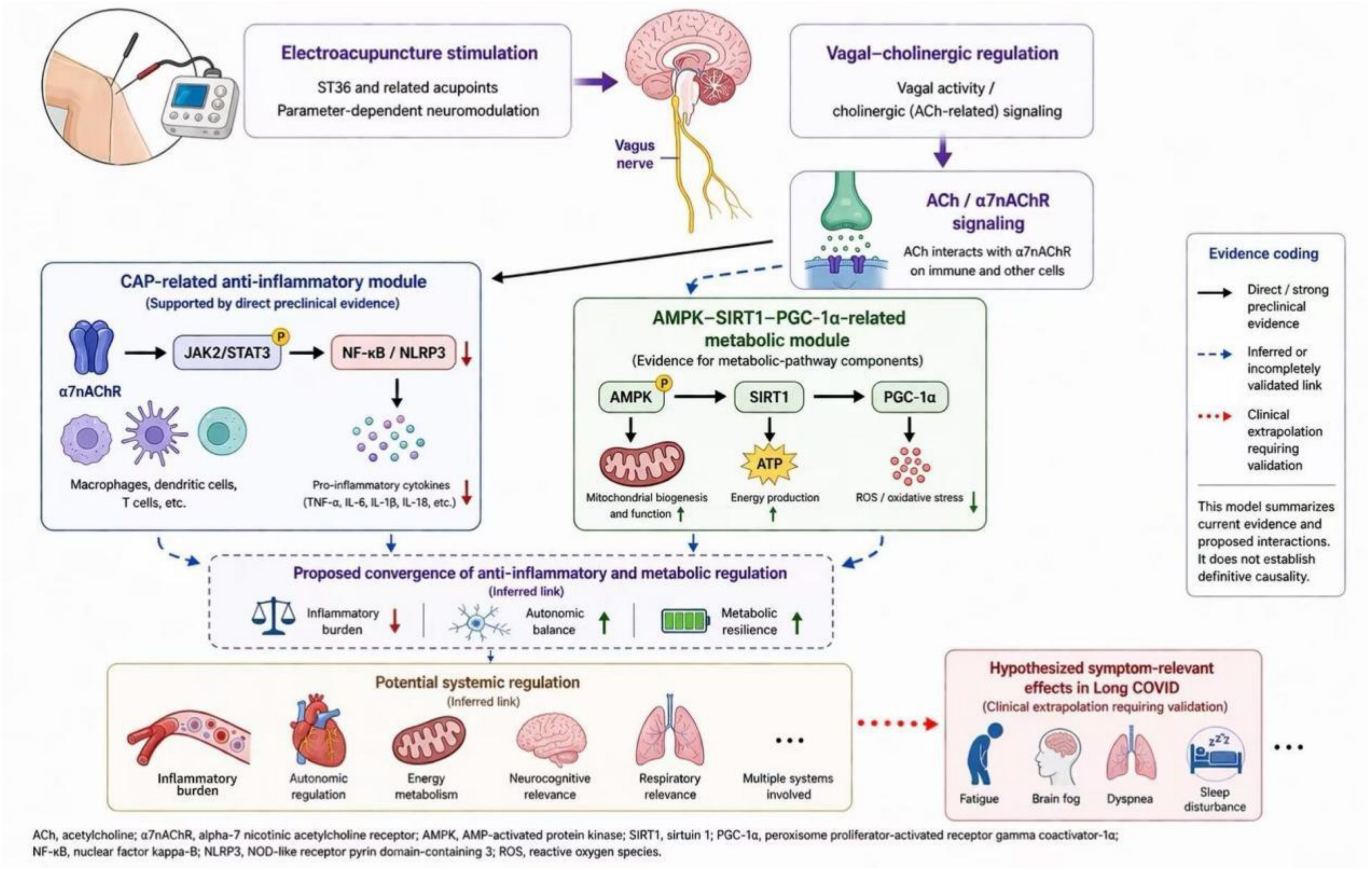
Evidence-graded EA–CAP–AMPK–SIRT1–PGC-1α regulatory model. This figure presents an evidence-informed model linking EA stimulation with vagal–cholinergic anti-inflammatory regulation and metabolic signaling. Black solid arrows indicate links supported by direct or strong preclinical evidence. Blue dashed arrows indicate inferred or incompletely validated regulatory links, including the proposed convergence between CAP-related anti-inflammatory signaling and AMPK–SIRT1–PGC-1α-related metabolic regulation. Red dotted arrows indicate clinical extrapolation from potential systemic regulation to symptom-relevant effects in long COVID that requires further validation. This model should be interpreted as a hypothesis-generating framework rather than a fully established causal pathway.

Supported by convergent evidence across multiple research layers, this study proposes an evidence-graded EA–CAP–AMPK–SIRT1–PGC-1α integrated mechanistic model (Figure 5). This model is intended to organize evidence-supported regulatory links and explicitly labeled inferred links within a unified framework, thereby explaining how EA-related vagal–cholinergic regulation may connect inflammatory control with metabolic regulation.

Within the evidence-supported component of the model, EA stimulation engages vagal–cholinergic signaling, and ACh-related activation of α7nAChR suppresses NF-κB/NLRP3-mediated inflammatory signaling, thereby reducing pro-inflammatory cytokine production (Lei and Duan, 2021). In parallel, EA-related metabolic effects involving the AMPK–SIRT1–PGC-1α axis have been reported in preclinical metabolic models, supporting the relevance of this pathway to mitochondrial bioenergetics, antioxidant defense, and cellular energy regulation (Li C. et al., 2025; Zhu et al., 2025).

The key integrative element of this model is the proposed regulatory convergence between CAP-related anti-inflammatory signaling and AMPK–SIRT1–PGC-1α-mediated metabolic regulation. This convergence is biologically plausible because inflammatory signaling and mitochondrial metabolism are tightly interconnected in chronic inflammatory states. However, whether EA-induced CAP activation directly drives AMPK–SIRT1–PGC-1α signaling remains incompletely established. Therefore, this connection is explicitly presented as an inferred regulatory link in Figure 5 rather than as a confirmed causal pathway.

The model comprises two interrelated pathways:

(1) Neuro-regulatory pathway:

EA → vagal–cholinergic regulation → ACh/α7nAChR signaling →α7nAChR–JAK2–STAT3/NF-κB axis → inhibition of inflammatory signaling.

(2) Metabolic pathway:

EA-related metabolic regulation → AMPK–SIRT1–PGC-1α axis → mitochondrial function support → reduced ROS → improved metabolic stability.

Together, these pathways provide a structured explanation for how EA may exert coordinated anti-inflammatory and metabolic regulatory effects. The innovation of this model lies in integrating EA-mediated neuroimmune and metabolic effects into a single evidence-graded framework, while distinguishing directly supported mechanisms from inferred regulatory convergence and clinical extrapolation. This framework provides a basis for future mechanistic experiments and phenotype-aware clinical studies in long COVID and related chronic inflammatory conditions.

### 5.3 Neural resonance modulation hypothesis

Although vagus nerve-mediated CAP provides an important framework for understanding the anti-inflammatory effects of EA, reflex-based models alone may not fully explain the effects of EA on multisystem symptoms, circadian regulation, and neuro-immune-metabolic integration. Based on a synthesis of current evidence, this paper proposes the NRM hypothesis as a conceptual extension of the EA-CAP framework.

The NRM hypothesis suggests that electroacupuncture may act through frequency-dependent somatosensory inputs that participate in central autonomic regulatory networks, including the NTS-DMV-hypothalamic and brainstem-limbic circuits. This concept is supported by neuroimaging studies, which indicate that acupuncture or electroacupuncture can modulate brain regions involved in autonomic, limbic, sensory, and cognitive regulation (Chen et al., 2025; Huang et al., 2022; Rodrigues et al., 2023; Zhou et al., 2024). Under specific stimulation conditions, these inputs may induce rhythmic coordination within central autonomic networks, thereby modulating vagal output and CAP-related inflammatory regulation. Evidence from HRV and electrophysiological studies further suggests that electrical stimulation of acupoints can elicit frequency-dependent autonomic or neurophysiological responses (Jia et al., 2011; Mayor et al., 2025).

These findings provide indirect neuroimaging and electrophysiological support for the plausibility of the NRM, but they do not directly prove the resonance mechanism. Therefore, the NRM should be interpreted as a testable hypothesis linking stimulation parameters, central rhythm modulation, vagal-cholinergic output, and downstream neuroimmune effects. Future studies should employ multimodal neurophysiological approaches to test this model, including fMRI, EEG–fNIRS, or animal local field potential recordings, in conjunction with HRV, ACh/ChE, α7nAChR, and inflammatory markers. Frequency-response comparisons may help determine whether specific ranges of EA parameters are associated with stronger autonomic or CAP-related responses.

Therefore, the NRM hypothesis should be viewed as a hypothesis-generating framework, whose value lies in proposing how to integrate stimulation frequency, central autonomic neural network dynamics, and vagal-cholinergic regulation into future mechanistic studies of electroacupuncture in long COVID and related chronic inflammatory conditions.

### 5.4 System integration: from inflammation suppression to homeostatic reconstruction

**FIGURE 6:**
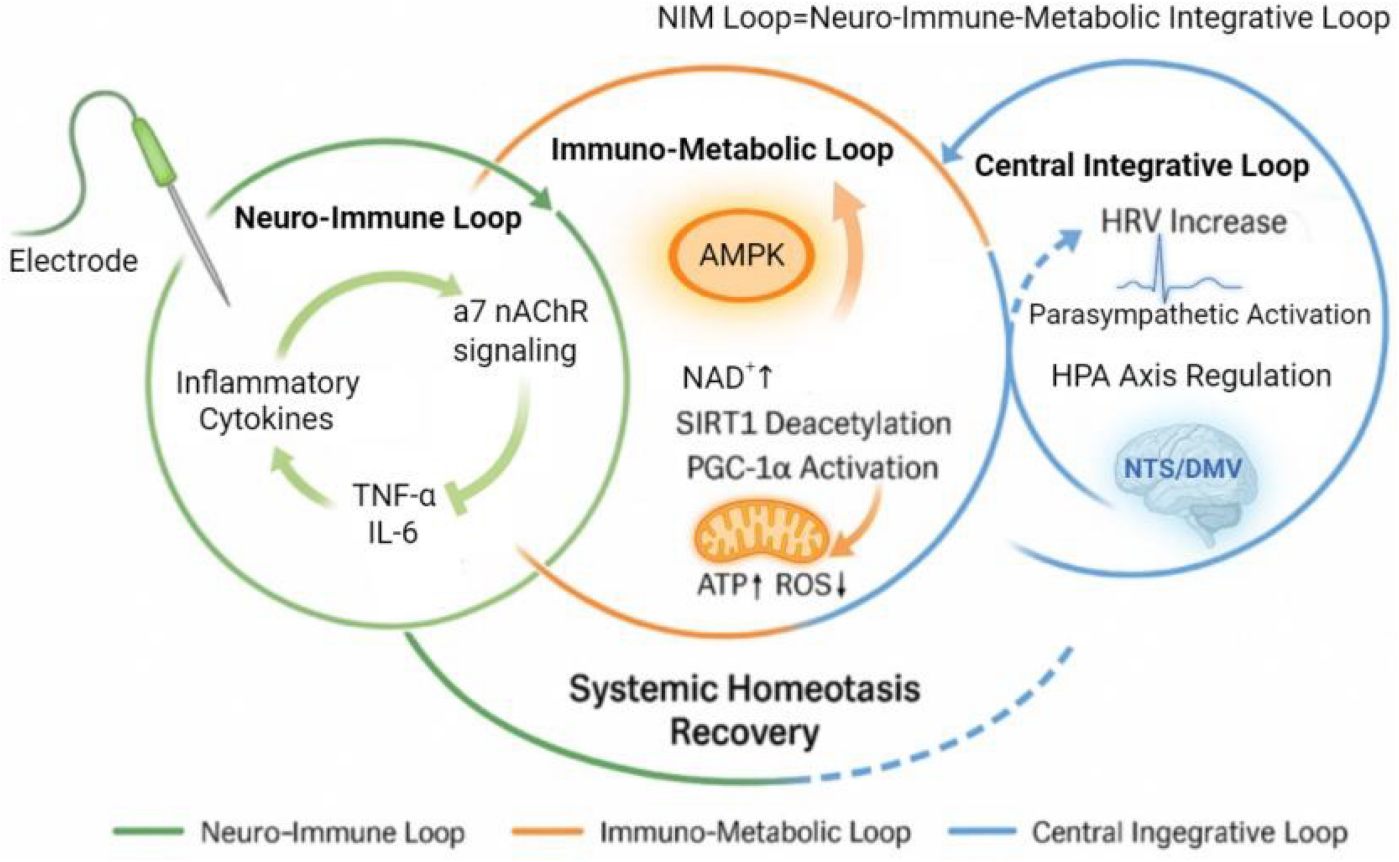
NIM Loops underlying EA-driven systemic recovery. This diagram outlines the mechanism by which EA promotes systemic recovery from long COVID through three interconnected loops. The neuro-immune loop is initiated by activating vagus nerve and cholinergic signaling, which act on α7nAChR receptors on immune cells to suppress the secretion of pro-inflammatory cytokines such as TNF-α and IL-6. The immune-metabolic circuit links this anti-inflammatory response to metabolic regulation by activating mechanisms that improve mitochondrial function mediated by SIRT1 and PGC-1α (manifested as increased ATP production and reduced reactive oxygen species). The core integration circuit on the far right involves the NTS-DMV pathway and broader autonomic-hypothalamic regulatory mechanisms, which contribute to increased parasympathetic tone, enhanced HRV, and stabilized HPA axis activity. Collectively, these circuits form the NIM integration network, further supporting the concept that acupuncture at specific points can facilitate systemic homeostasis restoration.

Integrating the above mechanisms, this study proposes the NIM loop, comprising three interactive layers (Figure 6):

(1) Neuro–Immune Loop

EA can activate the vagus nerve reflex arc, enhance CAP feedback, and simultaneously suppress the release of inflammatory cytokines, representing one pathway through which CAP exerts its anti-inflammatory effects.

(2) Immuno–Metabolic Loop

The α7nAChR signaling pathway may interact with AMPK–SIRT1–PGC-1α-related metabolic regulation, potentially supporting mitochondrial metabolism and antioxidant defense. This pathway serves as a pivotal hub linking inflammatory regulation and energy recovery in the body.

(3) Central Integrative Loop

The NRM-driven NTS-DMV-hypothalamic network synchronization mechanism restores autonomic balance and coordination with the HPA axis, thereby achieving top-down integration of the nervous, endocrine, and immune systems.

These neural, immune, and metabolic processes exhibit certain interrelationships. We can conceptualize them as three interwoven regulatory circuits, as illustrated in Figure 6.

These three circuits collectively form the NIM circuit, reflecting a hierarchical progression from suppressing inflammation to restoring systemic homeostasis.

This framework helps explain the improvement in symptoms such as fatigue and shortness of breath observed in patients with long COVID, as mentioned earlier. It also provides a feasible and scalable mechanism for immune activation therapies in other chronic inflammatory diseases.

### 5.5 Summary: innovation and mechanistic significance

Based on the interpretation of existing data, EA influences the neuro-immune-metabolic network through multiple interconnected steps rather than a single linear mechanism. Peripheral stimulation represented by EA activates the vagus nerve pathway, triggering CAP reflex activity and α7nAChR signaling. Subsequently, AMPK-SIRT1-PGC-1α-related metabolic signaling may become involved, exerting control over inflammation and metabolic regulation, ultimately achieving systemic physiological stability. While previous studies often simplify this process into a clear-cut framework, we prefer to view it as a dynamically interactive process rather than a singular chain of mechanisms.

**FIGURE 7:**
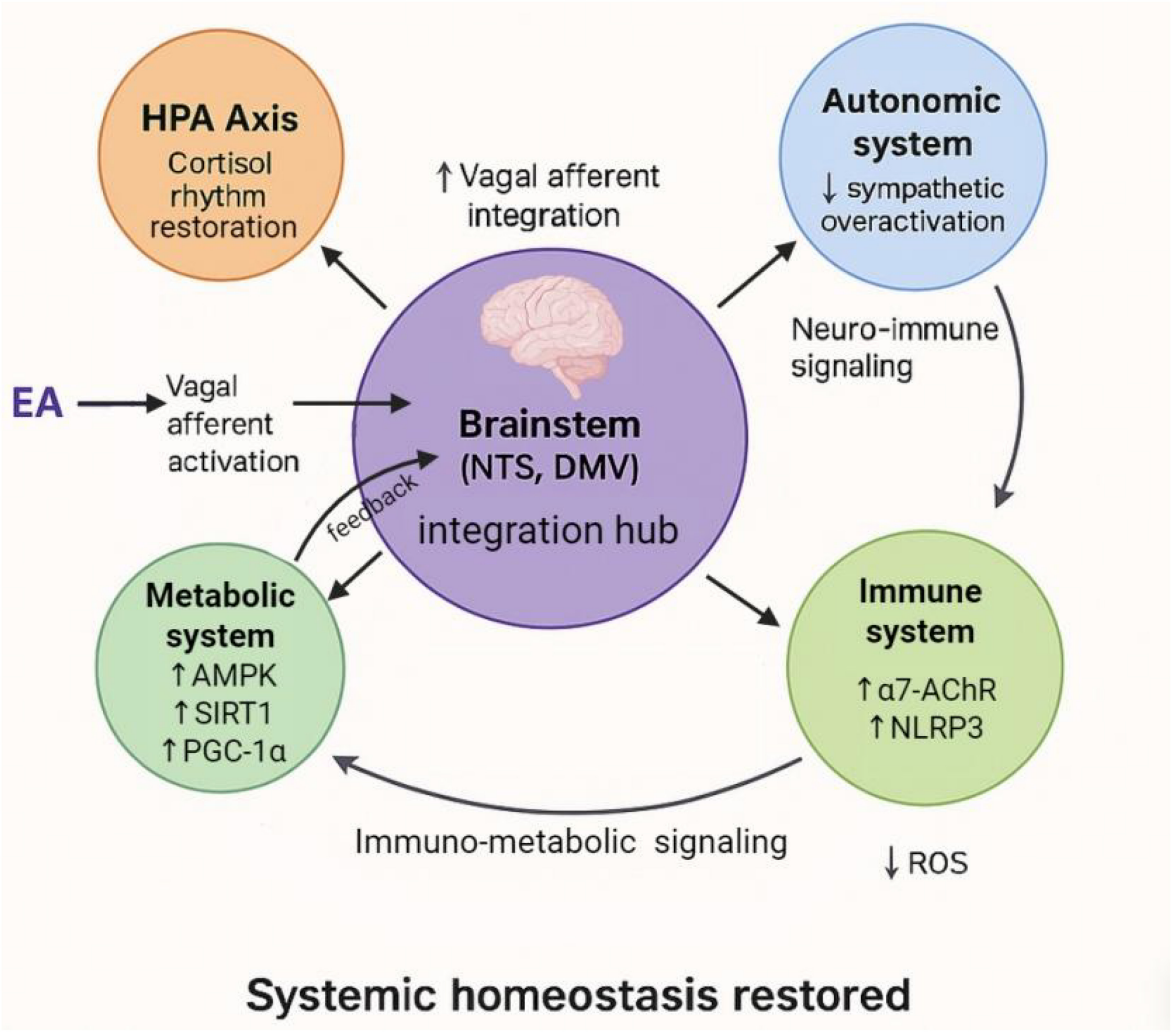
System-level integration of EA effects via neural–immune–metabolic resonance. This diagram summarizes at the systemic level how EA coordinates autonomic nervous, immune, metabolic, and neuroendocrine functions. Vagus nerve signals interact with brainstem hubs to regulate sympathetic-parasympathetic balance, suppress inflammatory responses, and enhance mitochondrial efficiency. Its synchronizing effect on the HPA axis promotes neuroendocrine stability. The synergistic interactions depicted reflect resonance-based mechanisms—multiple systems working in concert to restore homeostatic equilibrium. This diagram illustrates the synergistic effects of EA regulation across multiple physiological domains, serving as a model for our initial mechanistic research approach.

Based on these perspectives, we propose the EA-CAP-AMPK-SIRT1-PGC-1α integrated model and the NRM hypothesis as a framework to elucidate how EA coordinates the relationship between neural activity and immune metabolism. We depict these concepts as a NIM loop, which offers a more systematic perspective on EA’s mechanisms of action. This framework expands the discussion beyond anti-inflammatory regulation to encompass multi-level control, exploring how the body restores homeostasis through multiple regulatory layers. Figure 7 illustrates the integrated mechanisms of EA-induced neural, immune, and metabolic regulation at the systems level.

## 6 Future research and translational perspectives

Long COVID exhibits complex features of inflammation, autonomic dysfunction, and metabolic abnormalities, many of which point to impaired vagus nerve-cardiopulmonary axis signaling and disrupted neuro-immune-metabolic interactions. Based on the integrated mechanistic framework and evidence presented in this review, future research and clinical translation may consider the following directions:

### 6.1 Phenotype-oriented stratification and candidate operational criteria

Based on current clinical evidence and available guidelines, the identification of long COVID continues to rely primarily on a history of severe acute respiratory syndrome coronavirus 2 (SARS-CoV-2) infection, persistent symptoms lasting ≥ 3 months, and the exclusion of alternative diagnoses (Greenhalgh et al., 2024; Soriano et al., 2022). In the absence of definitive biomarkers, supplementary assessments—including autonomic function testing, analysis of inflammatory and metabolic biomarkers, and standardized symptom scales—may help characterize the key pathophysiological features of individual patients.

The following criteria may serve as candidate operational standards for phenotypic stratification but do not currently constitute formal diagnostic criteria. They are intended to aid in patient selection, subgroup analysis, and the design of future clinical trials. Thresholds should be adapted to local laboratory reference ranges and validated prospectively.

Common clinical assessment domains include HRV for autonomic regulation; inflammatory markers such as CRP, IL-6, and TNF-α; orthostatic testing for POTS-like autonomic instability; metabolic or oxidative stress markers such as lactate, ATP, SOD, and GSH-Px; and symptom scales such as FSS, PSQI, and the Montreal Cognitive Assessment (MoCA) (Buysse et al., 1989; Krupp et al., 1989; Lai et al., 2023; Nasreddine et al., 2005; Pearson et al., 2003; Sheldon et al., 2015; Soler-López et al., 2024). Based on these domains, four candidate long COVID phenotypes are proposed in Table 5.

**TABLE 5:** Candidate operational criteria for phenotype-oriented stratification of long COVID.

Phenotype	Core clinical features	Candidate inclusion criteria	Suggested threshold values	Exclusion/caution	EA translational focus
Autonomic-dominant phenotype	Palpitations, orthostatic intolerance, dizziness, sweating abnormality, sleep disturbance, anxiety-like autonomic symptoms, POTS-like presentation	Long COVID symptoms ≥ 3 months; prominent autonomic symptoms; abnormal HRV or orthostatic testing	HR increase ≥ 30 bpm within 10 min of standing/tilt without orthostatic hypotension; RMSSD or HF-HRV below age-adjusted reference; LF/HF ratio elevated; resting HR > 100 bpm as supportive criterion (Sheldon et al., 2015)	Exclude arrhythmia, hyperthyroidism, anemia, dehydration, medication-induced tachycardia, structural heart disease, and primary anxiety disorder	Prioritize vagal/autonomic outcomes: HRV, orthostatic HR response, COMPASS-31, sleep and autonomic symptom scales
Inflammatory-immune phenotype	Fatigue, myalgia, sore throat, inflammatory pain, feverish sensation, post-exertional symptom worsening	Long COVID symptoms ≥ 3 months; at least two inflammation-related symptoms; evidence of persistent inflammatory activation	hs-CRP > 3 mg/L or CRP above local upper reference range; IL-6, TNF-α, or IL-1β above local reference range; elevated neutrophil-to-lymphocyte ratio as a supportive criterion (Pearson et al., 2003)	Exclude acute infection, autoimmune flare, malignancy, uncontrolled inflammatory disease, recent vaccination, or acute febrile illness	Prioritize CAP-related anti-inflammatory outcomes: CRP, cytokines, α7nAChR-related markers, symptom burden
Metabolic-mitochondrial phenotype	Persistent fatigue, exercise intolerance, post-exertional malaise, low energy, muscle weakness, cognitive slowing	Long COVID symptoms ≥ 3 months; fatigue or exercise intolerance as dominant complaint; evidence of metabolic or mitochondrial stress if available	FSS mean score ≥ 4 or Chalder Fatigue Scale above validated cutoff; reduced 6-min walk distance or abnormal CPET when available; abnormal lactate response after exertion or delayed recovery; ATP, ROS, AMPK/SIRT1/PGC-1α-related markers as exploratory indicators (Krupp et al., 1989)	Exclude uncontrolled diabetes, hypothyroidism, severe anemia, sleep apnea, major depressive episode as primary cause, and cardiopulmonary limitation unrelated to long COVID	Prioritize metabolic outcomes: fatigue scale, 6-min walk test, CPET variables, lactate recovery, ATP/ROS/ mitochondrial markers
Mixed neuro–immune–metabolic phenotype	Multisystem symptoms involving fatigue, autonomic instability, inflammatory symptoms, cognitive complaints, sleep disturbance, and exercise intolerance	Long COVID symptoms ≥ 3 months; overlapping features from at least two of the above phenotypes; multi-domain abnormality on symptom scales or biomarkers	Meets partial criteria for ≥ 2 phenotype domains; abnormalities in ≥ 2 domains among HRV/orthostatic testing, inflammatory markers, fatigue scales, sleep/cognitive scales, or metabolic markers; PSQI > 5 or MoCA < 26 may be used as supportive criteria when sleep or cognitive symptoms dominate (Buysse et al., 1989; Nasreddine et al., 2005)	Avoid overclassification; exclude dominant alternative diagnoses and severe organ-specific disease requiring specialist management	Prioritize integrated outcomes: HRV, inflammatory markers, fatigue and sleep scales, cognitive testing, metabolic indicators

These criteria are proposed for research stratification and trial enrichment rather than formal diagnosis. Biomarker thresholds should be interpreted according to local laboratory reference ranges. HRV, heart rate variability; HF, high frequency; RMSSD, root mean square of successive differences; LF/HF, low-frequency/high-frequency ratio; POTS, postural orthostatic tachycardia syndrome; hs-CRP, high-sensitivity C-reactive protein; FSS, Fatigue Severity Scale; CPET, cardiopulmonary exercise testing; COMPASS-31, Composite Autonomic Symptom Score 31; ATP, adenosine triphosphate; ROS, reactive oxygen species; AMPK, AMP-activated protein kinase; SIRT1, sirtuin 1; PGC-1α, peroxisome proliferator-activated receptor gamma coactivator-1α.

These phenotypes are not mutually exclusive. Many patients may meet criteria for more than one phenotype, reflecting the overlapping nature of inflammation, autonomic dysregulation, metabolic impairment, and neurocognitive disturbance in long COVID. For clinical studies, patients may be assigned according to the dominant phenotype or stratified using composite scores. This approach may improve the interpretability of EA trials by linking stimulation protocols and outcome measures to specific pathophysiological profiles. These subtypes should therefore be interpreted as trial-enrichment categories rather than established clinical phenotypes.

### 6.2 Translational potential and key challenges in EA research

Increasing evidence indicates that transcutaneous electrical nerve stimulation activates the vagus nerve-mediated CAP, suppressing inflammatory responses, enhancing metabolic homeostasis, and promoting synergistic regulation of the neuro-immune-metabolic system (Huang et al., 2025; Zhang L. et al., 2021). These findings highlight the translational potential of EA in treating chronic inflammation and autonomic imbalance-related disorders, including post-COVID-19 sequelae.

However, several challenges remain. Current studies exhibit significant variations in stimulation parameters and acupoint combinations, limiting the reproducibility of results and cross-study comparability (MacPherson et al., 2010; Nielsen et al., 2024; Yu et al., 2025). Clinical assessments of acupuncture activation also lack standardized metrics; indicators such as HRV or circulating ACh are indirect measurements and lack specificity (Alen, 2022; Gitler et al., 2025). Mechanistic studies often focus on single pathways rather than validating the multi-layered neuro-immuno-metabolic coupling mechanisms proposed in this paper as potential underpinnings of acupuncture’s systemic effects (Fan, 2023; Zhang Q. et al., 2023).

Progress will require establishing CAP-centered quantitative assessment systems that integrate HRV, immunomics, metabolomics, and functional neuroimaging. Well-designed RCTs that incorporate CAP-related phenotyping may clarify whether EA exerts differential effects across distinct long COVID subgroups. To improve translational specificity, future EA studies should align acupoint selection, stimulation parameters, and outcome measures with the dominant long COVID phenotype. A phenotype-oriented translational strategy is summarized in Table 6.

**TABLE 6:** Phenotype-oriented EA translation strategies for long COVID.

Phenotype	EA strategy	Candidate acupoints	Stimulation parameters	Primary outcomes	Trial considerations
Autonomic-dominant phenotype	Vagal and autonomic regulation	ST36, PC6, HT7; auricular vagal-related points may be considered	Low-frequency EA, e.g., 2–15 Hz; 20–30 min/session; 2–3 sessions/week	HRV, orthostatic HR response, COMPASS-31, sleep/autonomic symptom scales	Enrich patients with abnormal HRV, orthostatic intolerance, or POTS-like features
Inflammatory-immune phenotype	CAP-oriented anti-inflammatory regulation	ST36, LI4, LI11, SP6	Low-frequency EA; parameters should be standardized and fully reported	CRP, IL-6, TNF-α, IL-1β, α7nAChR-related markers, symptom burden	Include inflammatory marker-positive subgroup analysis; exclude acute infection or active inflammatory flare
Metabolic-mitochondrial phenotype	Fatigue- and metabolism-focused regulation	ST36, SP6, CV4/CV6; EX-B3 may be considered if metabolic outcomes are prioritized	Repeated sessions over ≥ 4–8 weeks; frequency and intensity should be predefined	FSS, 6-min walk test, CPET variables, lactate recovery, ATP/ROS or mitochondrial markers	Enrich patients with fatigue, exercise intolerance, or metabolic stress indicators
Mixed neuro–immune–metabolic phenotype	Integrated multimodal regulation	ST36 + PC6/HT7, with individualized adjunct points according to dominant symptoms	Parameter selection guided by the dominant symptom domain; adaptive protocols may be considered	HRV, inflammatory markers, fatigue, sleep, cognition, and metabolic indicators	Use stratified or adaptive trial designs; avoid overclassification and prespecify composite outcomes

EA strategies are proposed for trial design and phenotype-oriented translational research rather than established treatment protocols. Acupoint selection, stimulation parameters, practitioner background, treatment regimen, control procedures, and intervention fidelity should be fully reported according to established acupuncture trial reporting standards and recent fidelity-monitoring recommendations (MacPherson et al., 2010; Nielsen et al., 2024). HRV, heart rate variability; CAP, cholinergic anti-inflammatory pathway; COMPASS-31, Composite Autonomic Symptom Score 31; CRP, C-reactive protein; FSS, Fatigue Severity Scale; CPET, cardiopulmonary exercise testing; ATP, adenosine triphosphate; ROS, reactive oxygen species.

### 6.3 Expanding clinical applications and directions for precision intervention

Future clinical applications of EA will likely depend on greater attention to standardization, individualization, and measurable physiological outcomes. Low-frequency EA (2–15 Hz) applied for 20–30 min, two to three times per week, remains a commonly used protocol in current practice (Cai et al., 2022; Li Q. et al., 2023). Acupoints such as ST36 and PC6, with the possible addition of LI4, HT7, or LR3, are often chosen to support autonomic balance and emotional regulation (Li L. et al., 2025).

Individualized strategies can be informed by physiological markers. Patients who present with low HRV or a sympathetic-dominant pattern may respond better to approaches that strengthen vagal activation, whereas individuals with metabolic or fatigue-predominant symptoms may require closer tracking of AMPK–SIRT1–PGC-1α–related metabolic indicators (Li L. et al., 2025; Zhang T. et al., 2021). Using multidimensional outcome measures—such as HRV, ACh, cytokines, fatigue scores, and cognitive or psychological indices—could facilitate the development of a quantitative CAP functional index for precision intervention.

Clinical trials related to long COVID are beginning to emerge. A recently published prospective, randomized, sham-controlled, patient-assessor-blinded trial protocol plans to evaluate electroacupuncture for long COVID neuropsychiatric symptoms. The study is designed to enroll 150 patients, with 32 EA sessions over 16 weeks and an 8-week follow-up, and includes cognitive, depressive, sleep, fatigue, and quality-of-life outcomes such as MMSE, the Chinese version of the Beck Depression Inventory, ISI, Brief Fatigue Inventory-Taiwan, and SF-12 (Wei D. J. et al., 2025) However, this is merely a study protocol and not conclusive evidence of efficacy; further trials of electroacupuncture based on phenotypic stratification are still required.

AI-assisted physiological monitoring may eventually support HRV-AI closed-loop neuromodulation systems capable of adjusting stimulation parameters in real time. Well-designed, phenotype-stratified randomized controlled trials are still needed to evaluate the clinical relevance of the proposed “EA–CAP–AMPK–SIRT1–PGC-1α framework” and to inform future translational guidance.

### 6.4 Multimodal research and AI integration

Recent advances in biosensors, wearable technologies, and machine-learning tools are beginning to shift EA research from fixed stimulation protocols toward data-driven neuromodulation. AI-assisted EA–neurofeedback systems may in future help track autonomic signals—such as HRV, EEG, or skin conductance—in real time, interpret individual CAP-related responses, and modify stimulation parameters accordingly. When viewed through the lens of the NRM hypothesis, these systems may also be capable of identifying oscillatory features within brainstem–hypothalamic circuits and adjusting EA inputs to better match central rhythmic activity, a strategy that could enhance treatment stability and broaden systemic regulatory effects. These advances collectively indicate a shift toward more digital and adaptive neuromodulation based on EA-based neural modulation in both experimental and clinical settings.

### 6.5 Summary

In long COVID, the effects of acupuncture appear to extend beyond local neural reflexes to broader interactions involving autonomic nervous system, immune, and metabolic regulation. By modulating CAP-related and metabolic signaling networks, acupuncture may help restore balance across multiple physiological systems. The EA-CAP-AMPK-SIRT1-PGC-1α integrated mechanism, combined with the neuromodulatory hypothesis and the NIM feedback loop, provides a clear and coherent explanatory framework for these effects. Taken as a whole, these concepts place EA within a growing systems-neuroscience and precision-medicine perspective and provide a platform for future translational and clinical work.

## 7 Conclusion

By synthesizing evidence from animal models, clinical observations, and studies in neuroimmunology and cellular energy regulation, this review highlights that the persistent inflammation and functional dysregulation observed in long COVID are unlikely to arise from a single pathological mechanism. Instead, convergent findings across disciplines point toward multi-level interactions among neural, immune, and metabolic processes as key contributors to symptom persistence. Within this context, modulation of autonomic nervous function—particularly vagal afferent and efferent activity—emerges as a recurring regulatory theme linking inflammatory control with cellular metabolic homeostasis.

At the central level, available experimental and neurophysiological studies suggest that electrical stimulation may influence rhythmic activity within brainstem–hypothalamic autonomic networks, including the nucleus tractus solitarius and dorsal motor nucleus of the vagus. To facilitate interpretation of these observations, this review employs conceptual descriptors such as Neural Resonance Modulation and broader neuro–immune–metabolic coupling to illustrate coordinated regulatory patterns, rather than to assert experimentally validated mechanistic entities. From this perspective, these frameworks function primarily as organizational tools that integrate heterogeneous findings across neural, immune, and metabolic domains.

Within this integrative framework, reduced responsiveness of the cholinergic anti-inflammatory pathway referred to here as “CAP hyporesponsiveness”, is discussed as a potential explanatory construct for recurrent features reported in some individuals with long COVID, including low vagal tone, insufficient inflammatory feedback regulation, and associated metabolic disturbance. Importantly, this concept is not intended to define a distinct clinical syndrome, but rather to summarize patterns observed across studies that warrant further investigation. Clarification of the underlying physiological relationships will require longitudinal designs, multimodal autonomic assessment, and targeted mechanistic research.

Looking forward, continued advances in electrophysiological monitoring, heart rate variability analysis, and integrated immunomic and metabolomic profiling are expected to enhance the experimental tractability of vagal regulation, cholinergic anti-inflammatory signaling, and neuro–immune–metabolic interactions. Although long COVID represents a timely and clinically relevant application context, the regulatory perspectives discussed in this review are likely to extend to a broader spectrum of chronic inflammatory and post-infectious conditions. In this sense, the present synthesis does not seek to establish definitive mechanisms, but rather to provide a structured and cautious framework that may guide future experimental and translational research on neuromodulatory interventions targeting complex physiological networks.
